# Acylation of the antimicrobial peptide CAMEL for cancer gene therapy

**DOI:** 10.1080/10717544.2020.1787556

**Published:** 2020-07-02

**Authors:** Jingjing Song, Panpan Ma, Sujie Huang, Juanli Wang, Huan Xie, Bo Jia, Wei Zhang

**Affiliations:** a The Institute of Pharmacology, Key Laboratory of Preclinical Study for New Drugs of Gansu Province, School of Basic Medical Sciences, Lanzhou University, Lanzhou, China; b Institute of Biochemistry and Molecular Biology, School of Life Sciences, Lanzhou University, Lanzhou, China; c Institute of Physiology, Key Laboratory of Preclinical Study for New Drugs of Gansu Province, School of Basic Medical Sciences, Lanzhou University, Lanzhou, China

**Keywords:** Antimicrobial peptide, non-viral vector, cancer gene therapy, p53, MDM2 inhibitor

## Abstract

Obtaining ideal gene delivery vectors is still a major goal in cancer gene therapy. CAMEL, a short hybrid antimicrobial peptide, can kill cancer cells by membrane lysis. In this study, we constructed a series of non-viral vectors by attaching fatty acids with different chain lengths to the N-terminus of CAMEL. Our results showed that the cellular uptake and transfection efficiency of acyl-CAMEL started to significantly increase from a chain length of 12 carbons. C18-CAMEL was screened for gene delivery because it had the highest transfection efficiency. Surprisingly, C18-CAMEL/plasmid complexes displayed strong endosomal escape activity after entering cells via endocytosis. Importantly, C18-CAMEL could deliver p53 plasmids to cancer cells and significantly inhibited cell proliferation by the expression of p53. In addition, the C18-CAMEL/p53 plasmid complexes and the MDM2 inhibitor nutlin-3a showed significantly synergistic anticancer activity against MCF-7 cells expressing wild-type p53. Conclusively, our study demonstrated that conjugation of stearic acid to antimicrobial peptides is a simple and successful approach for constructing efficient and economical non-viral vectors for cancer gene therapy.

## Introduction

The overall survival rate of cancer patients is still low despite advances in cancer treatment. Therefore, novel effective treatment strategies are urgently needed to improve cancer clinical outcomes. As progress has been made in the elucidation of the mechanisms of cancer development, cancer has been widely accepted as a genetic disease. Gene therapy based on the cellular uptake of therapeutic nucleic acids with different functions, such as the production of cytotoxic proteins, enhancement of the immune response, silencing of oncogene expression and genome editing, has shown promise in cancer treatment (Kullberg et al., 2013; Hill et al., 2016; Liu et al., 2017; Chen M et al., 2019; Roma-Rodrigues et al., 2020). Many viral and non-viral gene vectors have been developed for cancer therapy in recent decades (Kullberg et al., 2013; Zhou et al., 2017). Although viral vectors fulfill the criteria for a strong gene-delivery capacity, there are also several important drawbacks, such as immunogenicity, insertional mutagenesis, limited cargo capacity, and difficulty of vector production (Lehto et al., 2011; Lachelt & Wagner, 2015; Hill et al., 2016). These inherent limitations have led to the development of efficient non-viral vectors with improved safety profiles (Yin et al., 2014; Bono et al., 2020).

Cell-penetrating peptides (CPPs) are a group of short peptides and can be categorized based on their physicochemical properties into three main classes: cationic, amphipathic and hydrophobic CPPs (Milletti, 2012; Xu et al., 2019). CPPs have been widely employed to construct non-viral gene vectors due to their high cell-penetrating efficiency, ease of synthesis and functionalization and relatively low toxicity (Lehto et al., 2011; Nakase et al., 2012; Boisguerin et al., 2015; Taylor & Zahid, 2020). Modification with fatty acids, especially stearic acid, has proven to be a simple and successful strategy for enhancing the nucleic acid delivery efficiency of many CPPs (Nakase et al., 2012; Lehto et al., 2016). However, endosomal entrapment is still a crucial bottleneck that hampers the transfection efficiency of CPPs (Nakase et al., 2012; Lehto et al., 2016).

Antimicrobial peptides (AMPs) are short and cationic sequences that are similar to many CPPs in structure (Henriques et al., 2006). In addition to antimicrobial activity, a growing number of studies have indicated that AMPs display substantial cytotoxicity against cancer cells (Baxter et al., 2017; Hoskin & Ramamoorthy, 2008). Due to the special mechanism of membrane-lysis, many AMPs have been employed to facilitate the endosomal escape of non-viral gene vectors (Ferrer-Miralles et al., 2008; Hou et al., 2015). In addition, numerous AMPs were shown to translocate into cells like CPPs (Henriques et al., 2006; Splith & Neundorf, 2011). Based on these above characteristics of AMPs, we developed efficient non-viral vectors with high endosome-lytic activity by conjugating stearic acid to the antimicrobial peptide melittin (stearyl-Mel) and its retro isomer (stearyl-*r*Mel). These vectors, especially stearyl-*r*Mel, could deliver p53 plasmids into cancer cells and subsequently induce cell death (Zhang et al., 2013). However, the relatively long sequences of melittin and its retro isomer are not easy to synthesize and purify. Therefore, we want to seek more economical alternative vectors with satisfactory transfection efficiency.

CAMEL, a hybrid antimicrobial peptide containing residues 1–7 of cecropin and residues 2–9 of melittin, displayed potent antimicrobial activity without causing hemolysis (Andreu et al., 1992). In addition, CAMEL was reported to exhibit strong anticancer activity by disrupting mitochondria after translocation into cells (Smolarczyk et al., 2010). Compared with melittin containing 26 amino acids, CAMEL is a short antimicrobial peptide containing only 15 amino acids. Therefore, we suggest that CAMEL can become an ideal alternative for constructing an efficient non-viral gene vector for cancer therapy. In this study, non-viral vectors were constructed by attaching fatty acid groups, including butanoic acid (C4), octanoic acids (C8), lauric acid (C12), palmitic acid (C16) and stearic acid (C18), to the N-terminus of CAMEL. To obtain ideal non-viral vectors, we performed a series of experiments. In addition, the application potential of acyl-CAMEL in cancer gene therapy was also evaluated.

## Materials and methods

### Peptides synthesis

All peptides were synthesized using FMOC SPPS strategy. All the peptides were chemically synthesized manually using Fmoc chemistry on Rink amide MBHA resin. Briefly, the amino acid (3 equiv.) together with N-[(lH-benzotriazol-l-yl)(dimethylammo)methylene]-Nmethylmethanaminium hexafluorophosphate N-oxide (HBTU, 3 equiv.), 1-Hydroxybenzotriazole (HOBt, 3 equiv.) and N,N-Diisopropylethylamine (DIEA, 6 equiv.) in dimethylformamide (DMF) were coupled for 60 min. Fatty acids were coupled as amino acids. The Fmoc protecting group was removed by treatment with 20% piperidine in DMF. Following synthesis, the resin was washed with several portions of DMF, dichloromethane, and methanol before it was dried in a vacuum for at least 3 h. The final peptides were cleaved from the resin by treatment with a solution of trichloroacetic acid (TFA)/triisopropylsilane (TIS)/water (95:2.5:2.5, v/v/v) for 3 h at room temperature. TFA was removed by evaporation and the product precipitated in cold diethyl ether. Fractions were pooled and lyophilized. Acyl-peptides were synthesized by coupling the corresponding fatty acids to the N-terminus of peptide resins. After cleavage from the resin, the desired peptides were purified by reversed-phase high-performance liquid chromatography (RP-HPLC) on a C18 column. Purity analysis was checked by analytical RP-HPLC. The synthetic peptides were characterized by electrospray ionization-mass spectrometry (ESI-MS). The purity of all peptides used for experiments was ≥95%.

### Cell culture and amplification of plasmid DNA

COS-7, U87, U251, MCF-7, MB-MDA-231, Hela, Hepg2 and B16 cell lines were cultured in DMEM supplemented with 10% fetal bovine serum (FBS) in a 5% CO_2_ humidified atmosphere at 37 °C. All cell lines were obtained from the Chinese Academy of Sciences. pGL3 plasmid containing luciferase gene and pcDNA3.1 plasmid containing wild p53 gene were transformed in Escherichia coli DH5α and were amplified in LB medium at 37 °C overnight at 180 rpm. The plasmids were purified by an EndoFree Plasmid kit (TIANGEN, Beijing, China). Then the purified plasmids were dissolved in distilled water and stored at −20 °C.

### Complexes formation

All complexes were formed by mixing peptides and plasmids (0.5 μg) in 50 μL of water at various N/P ratios (ratio of positive charges of the peptide to negative charges of the plasmid) and were incubated for 30 min at 37 °C to form stable nanoparticles. Then, these complexes were diluted to a final volume of 500 μL and used immediately.

### *In vitro* transfections

Cells were seeded at 1 × 10^5^ cells/well in a 24-well plate 24 h before treatment. After washing with PBS, cells were treated with peptide/pGL3 plasmid complexes in 500 μL of DMEM containing FBS free or FBS at various concentrations for 4 h. Thereafter, the medium was replaced with 1 mL of DMEM containing 10% FBS. After incubation for 20 h, luciferase activity was measured using Promega’s luciferase detection kit. Data were normalized to protein content measured using a BCA protein assay kit (Pierce). Lipofectamine 2000 (LF2000, Invitrogen) served as a positive control. To evaluate the effect of chloroquine (CQ) on the transfection efficiency of C18-CAMEL, COS-7 cells were treated with peptides/pGL3 plasmid complexes and CQ (final concentration 100 μmol/L) for 4 h. After 20 h of incubation, luciferase activity was determined according to the above methods. Three independent experiments were performed.

### Cellular uptake assay

To quantify the cellular uptake of peptides/pGL3 plasmid complexes, COS-7 cells were cultured in a 24-well plate 24 h before treatment. After washing with PBS, cells were incubated with peptides/Cy5-labeled pGL3 plasmid complexes at an N/P ratio of 2. Labeling of the pGL3 plasmid with the fluorescent probe Cy5 was performed using a Label IT Tracker kit (Mirus), as described by the manufacturer. After 4 h of incubation, the cells were washed with PBS and then incubated with 0.02% trypsin for 10 min. The cells were harvested and centrifuged at 1500 rpm for 5 min. Thereafter, the cell pellets were resuspended and detected with a BD FACS Caliber Flow Cytometer. To explore the cellular uptake pathways of C18-CAMEL/pGL3 plasmid complexes, COS-7 cells were preincubated with an endocytosis inhibitor chlorpromazine (10 μg/mL), amiloride (50 μM), or methyl-β-cyclodextrin (5 mM) for 30 min, and then were incubated with peptide/Cy5-labeled pGL3 plasmid complexes at an N/P ratio of 2. After 4 h of incubation, the cellular uptake of complexes was detected using the above method. Three independent experiments were performed.

### Gel retardation assay

To explore the binding ability of CAMEL or C18-CAMEL with pDNA, peptides, and pGL3 plasmids were mixed at various ratios and at 37 °C for 30 min to form complexes. Thereafter, samples were electrophoresed through the 0.8% (W/V) agarose gel and imaged by staining the gel with EtBr.

### DNA condensation assay

The DNA condensation ability of peptides was evaluated using the ethidium bromide (EtBr) exclusion assay. pGL3 plasmids (0.5 μg) were mixed with peptides at various N/P ratios in 50 μL of milli-Q water. After incubation for 1 h, 135 μL of water was added to each sample and transferred into a 96-well black plate. Thereafter, 15 μL of EtBr solution was added to give a final EtBr concentration of 400 nmol/L. After 10 min of incubation, the 96-well plate was shaken orbitally for 30 s and the fluorescence intensity measured. Three independent experiments were performed.

### Transmission electron microscopy

TEM was used to observe the morphology of C18-CAMEL/pGL3 plasmid complexes at an N/P ratio of 2. C18-CAMEL/pGL3 plasmid complexes were prepared as described above. The TEM samples were prepared by dropping 10 μL of C18-CAMEL/pGL3 plasmid complex solution onto a copper grid and then staining by 0.2% (W/V) phosphotungstic acid solution before measurement.

### Particle size and ζ-potential measurements

C18-CAMEL/pGL3 plasmid complexes were prepared as described above. These complexes were diluted in phosphate-buffered saline (PBS) to 1 mL volume, and then, the size and ζ-potential were measured by Nano-ZS ZEN3600 (Malvern Instruments, Malvern, UK). Three independent experiments were performed.

### Antiproliferative assay

The antiproliferative effect of C18-CAMEL/p53 plasmid complexes was determined by the MTT assay. Cells were seeded in 96-well plates at a density of 5 × 10^3^ cells/well. After being washed, cells were treated with C18-CAMEL alone, p53 plasmid alone, and C18-CAMEL/p53 plasmid complexes in 100 μL of DMEM at various N/P ratios for 4 h. Thereafter, 100 μL of DMEM containing 10% FBS was added, and the cytotoxicity was determined by the MTT assay after 96 h. Three independent experiments were performed.

To study the antiproliferative effect of C18-CAMEL/p53 plasmid complexes in combination with MDM2 inhibitor nutlin-3a against different cell lines, cells were treated with C18-CAMEL/p53 plasmid complexes at an N/P ratio of 2, nutlin-3a at different concentrations, C18-CAMEL, a combination of C18-CAMEL and nutlin-3a, or combination of C18-CAMEL/p53 plasmid complexes and nutlin-3a in 100 μL of DMEM for 4 h. Thereafter, 100 μL of DMEM containing 10% FBS was added, and the cytotoxicity was determined by the MTT assay after 96 h. Three independent experiments were performed.

### P53 expression assay

For mRNA level analysis, after transfection with C18-CAMEL/p53 plasmid complexes for 4 h, MCF-7 cells were collected and total RNA was extracted using the TRIzol reagent. Approximately, 1 μg of total RNA from each sample was converted to complementary cDNA using a commercially available RT-PCR kit. The obtained cDNA was then employed as a template for classical PCR amplification as follows. The PCR products were detected by 2% agarose gel electrophoresis. The primers used were as follows: p53 sense 50-CCTCAGCATCTTATCCGAGTGG-30 and antisense 50-TGGATGGTGGTACAGTCAGAGC-30. Three independent experiments were performed.

For p53 protein expression level analysis, western blot analysis was performed. Briefly, MCF-7 cells were transfected with peptide/p53 plasmid complex for 48 h. Thereafter, cells were harvested and lysed, and protein concentrations were determined by using a BCA protein assay kit (Pierce). The total amount of 40 μg of protein from each sample was loaded and separated on a 10% SDS − PAGE gel. After electrophoresis, the samples were transferred onto a PVDF membrane. The membranes were probed with the primary antibody specific for p53 followed by incubation with the HRP-conjugated secondary antibody. The signal was detected by an enhanced chemiluminescence detection system.

### Statistical analysis

Experiments were performed three times and the data were expressed as means ± SEM. Statistical analysis was performed using Student’s *t*-tests. *p* < .05 was considered to be indicative of statistical significance.

## Results and discussion

### Transfection efficiency of peptides

Delivery of therapeutic nucleic acids with poor bioavailability into cells is a prerequisite for cancer gene treatment. Recently, CPP-based vectors have attracted considerable interest as non-viral vectors due to their efficiency (Lehto et al., 2016; Sun et al., 2017). Given that AMPs share common features with CPPs in various aspects, such as physicochemical characteristics and strong membrane association, AMPs may provide us with inspiration to design more efficient gene vectors (Henriques et al., 2006; Splith & Neundorf, 2011). CAMEL is a hybrid antimicrobial peptide with membrane-lysis and cell-penetrating activity (Smolarczyk et al., 2010). It was reported that the attachment of fatty acids with different chain lengths can influence the transfection efficiency of acyl-CPPs (Katayama et al., 2011; Lehto et al., 2017; Morais et al., 2018). Therefore, a series of vectors were synthesized by attaching fatty acids with different chain lengths to the N-terminus of CAMEL in the present study (Table 1).

**Table 1.** Peptide sequences.

Peptide	Sequences	MW_cal_	MW_obs_
CAMEL	KWKLFKKIGAVLKVL-NH_2_	1769.2	1770.2
C4-CAMEL	CH_3_-(CH2)_2_-CO-KWKLFKKIGAVLKVL-NH_2_	1839.4	1840.3
C8-CAMEL	CH_3_-(CH2)_6_-CO-KWKLFKKIGAVLKVL-NH_2_	1895.5	1896.3
C12-CAMEL	CH_3_-(CH2)_10_-CO-KWKLFKKIGAVLKVL-NH_2_	1951.6	1951.7
C16-CAMEL	CH_3_-(CH2)_14_-CO-KWKLFKKIGAVLKVL-NH_2_	2007.7	2008.5
C18-CAMEL	CH_3_-(CH2)_16_-CO-KWKLFKKIGAVLKVL-NH_2_	2035.8	2036.5
*r*CAMEL	LVKLVAGIKKFLKWK-NH_2_	1769.3	1770.7
C12-*r*CAMEL	CH_3_-(CH2)_10_-CO-LVKLVAGIKKFLKWK-NH_2_	1951.6	1952.3
C16-*r*CAMEL	CH_3_-(CH2)_14_-CO-LVKLVAGIKKFLKWK-NH_2_	2007.7	2008.4
C18-*r*CAMEL	CH_3_-(CH2)_16_-CO-LVKLVAGIKKFLKWK-NH_2_	2035.8	2036.5
stearyl-*r*Mel	CH_3_-(CH2)_16_-CO-QQRKRKIWSILAPLGTTLVKLVAGIG-NH_2_	3111.0	3111.9
C18-NTAT	CH_3_-(CH2)_16_-CO-PKKKRKVYGRKKRRQRRR-NH_2_	2688.5	2689.8

**Figure 1.**
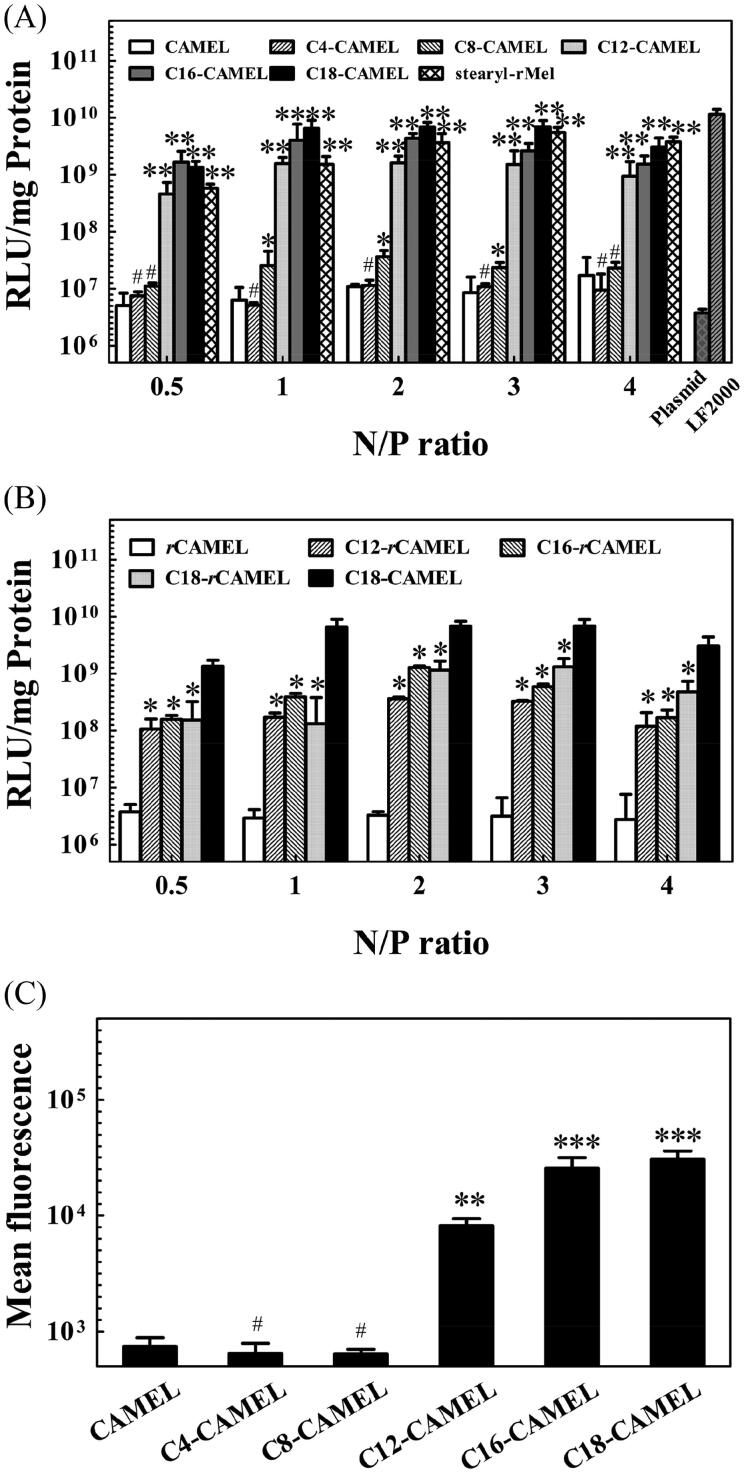
Comparative study of transfection efficiency of peptides in COS-7 cells. (A) Luciferase expression of cells treated with acyl-CAMEL/pGL3 plasmid complexes. Stearyl-*r*Mel and LF2000 served as the control. #*p* > .05 versus CAMEL; *p < .05 versus CAMEL; **p < .01 versus CAMEL. (B) Luciferase expression of treated with acyl-*r*CAMEL/pGL3 plasmid complexes. *p < .05 versus C18-CAMEL. (C) Cellular uptake efficiency of acyl-CAMEL/Cy5-labeled plasmids complexes at an N/P ratio of 2. #*p* > .05 versus CAMEL; **p < .01 versus CAMEL; ***p < .001 versus CAMEL.

To identify satisfactory non-viral vectors for cancer gene therapy, we first evaluated the transfection efficiency of CAMEL and acyl-CAMEL in COS-7 cells using pGL3 plasmid, which contains a reporter gene encoding luciferase. As shown in Figure 1(A), the luciferase expression in the COS-7 cells transfected with the CAMEL/plasmid complexes, the C4-CAMEL/plasmid complexes, and the C8-CAMEL/plasmid complexes displayed a slight increase over that of the naked plasmids at various N/P ratios. When the chain length exceeded 12 carbons, the transfection efficiency of C12, C16, and C18-CAMEL showed a strong increase. Among these vectors, C18-CAMEL, with the highest hydrophobicity, displayed the strongest transfection efficiency with Lipofectamine 2000 (LF2000). Our results are consistent with a previous study, where the transfection of acyl-peptides started to pronouncedly increase from a chain length of 12 carbons (Lehto et al., 2017). Importantly, the transfection efficiency of C18-CAMEL was higher than that of stearyl-*r*Mel, which is constructed by stearylation of the retro isomer of antimicrobial peptide melittin and was reported to display high transfection efficiency in our previous study (Zhang et al., 2013). In addition, we unexpectedly found that stearyl-*r*Mel with a reverse sequence exhibited approximately 10-fold transfection efficiency compared with stearyl-Mel. Therefore, we also constructed vectors by attaching C12, C16, and C18 to the N-terminus of the retro isomer of CAMEL (Table 1). Unfortunately, the transfection efficiency of C12-, C16- and C18-*r*CAMEL was substantially lower than that of C18-CAMEL (Figure 1(B)). This result demonstrates that this strategy is not suitable for all peptides to enhance their transfection efficiency.

To further address the impact of fatty acids with different chain lengths on the transfection efficiency of acyl-CAMEL, we used FACS to evaluate the cellular uptake efficiency of the complexes formed by acyl-CAMEL and Cy5-labeled pGL3 plasmids at an N/P ratio of 2. As shown in Figure 1(C), the fluorescence intensity of the internalized complexes started to increase from an acyl chain length of 12 carbons. This result demonstrated that the vectors based on CAMEL with longer carbon chain lengths displayed higher plasmid delivery efficiency, strongly supporting the result derived from the transfection efficiency assay.

Taken together, the above results indicated that C18-CAMEL displayed the highest transfection efficiency among these vectors. Thus, in the following studies, we mainly evaluated the application potential of C18-CAMEL for cancer gene therapy.

### Characterization of the peptide/plasmid complexes

A non-covalent strategy has proven to be a simple, cost-efficient, and effective methodology for CPP-based vectors to deliver nucleic acids into cells (Deshayes et al., 2008). This strategy predominantly relies on electrostatic- and hydrophobic interactions, which can facilitate cationic CPPs to condense anionic nucleic acids into more stable nanoparticles (Deshayes et al., 2008; Lehto et al., 2011). Attachment of fatty acids is an effective approach for many CPPs to efficiently condense nucleic acids (Lehto et al., 2011; Nakase et al., 2012; Lehto et al., 2016). Many factors, such as nucleic acid condensation, size, zeta potential, and shape, were reported to influence the transfection efficiency of nanoparticles formed by CPPs and nucleic acids (Wang et al., 2011; Sharma et al., 2013; Dutta et al., 2015; Lehto et al., 2017; Suchaoin et al., 2017). Therefore, we explored these physicochemical characteristics of C18-CAMEL/pGL3 plasmid complexes.

**Figure 2.**
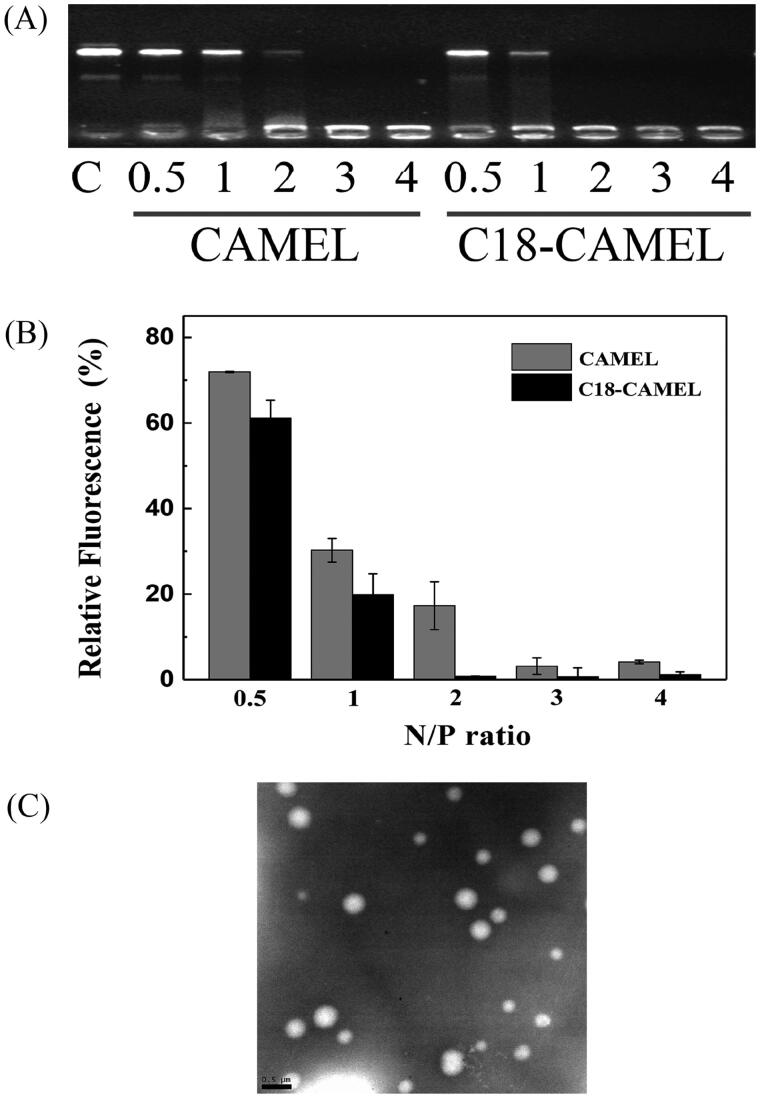
Characterization of C18-CAMEL/pGL3 plasmid complexes. (A) Gel retardation assay of DNA binding ability of the C18-CAMEL. (B) Ethidium bromide exclusion assay of DNA binding ability of the C18-CAMEL. (C) TEM micrograph of C18-CAMEL/pGL3 plasmid complexes at an N/P ratio of 2. Bar, 0.5 μm.

To evaluate the extent to which peptides are capable of binding and condensing pDNA, we performed a gel retardation assay and ethidium bromide (EtBr) exclusion assay. As shown in Figure 2(A), the results derived from the gel retardation assay indicated that CAMEL completely retarded pDNA migration at an N/P ratio of 3, while C18-CAMEL exhibited the same pDNA binding capacity at an N/P ratio of 2. This result demonstrated that C18-CAMEL exhibits a stronger pDNA binding capacity than CAMEL. In addition, at N/P ratios of 3 and 4, the brightness of the pDNA bands in the sample wells containing C18-CAMEL/pDNA complexes was substantially lower than that in the sample wells containing CAMEL/plasmid complexes. These results implied that C18-CAMEL and pDNA could form stable complexes that can prevent EtBr interaction with pDNA (McCarthy et al., 2014). The results derived from the EtBr exclusion assay also showed that unmodified CAMEL condensed pDNA to a lower extent than C18-CAMEL (Figure 2(B)). This result coincided with that of the gel retardation assay, demonstrating that N-terminal stearylation can efficiently facilitate the pDNA binding and condensing capacity of CAMEL.

The size and zeta potentials of CPPs/pDNA complexes are critical features for efficient transfection. It was reported that the size of the complexes should not exceed 300 nm for efficient internalization (Hoyer & Neundorf, 2012). In this study, our results showed that the size of the C18-CAMEL/pDNA complexes at various N/P ratios was in the range of 190–260 nm (Table 2). Furthermore, TEM images revealed that C18-CAMEL/pDNA complexes at an N/P ratio of 2 displayed a spherical shape with smooth edges (Figure 2(C)), confirming that C18-CAMEL can indeed compact pDNA into regular nanoparticles. The zeta potential of the C18-CAMEL/pDNA complexes showed a negative value (−20.6 mV) at an N/P ratio of 1, while it increased to positive values ranging from 11.8 to 18.5 mV at an N/P ratio of 2 and greater (Table 2). The increased positive zeta potential values of the complexes mean that more cationic C18-CAMEL is engaged in pDNA binding, resulting in forming more stable nanoparticles. On the other hand, the increased zeta potential values can facilitate the binding of complexes to negatively charged cell membranes and subsequent translocation. (Hoyer & Neundorf, 2012; Raucher & Ryu, 2015).

**Table 2.** Particle size, zeta potential, and polydispersity index of C18-CAMEL/pGL3 plasmid complexes.

N/P ratio	Particle size (nm)	Zeta potential (mV)	Polydispersity index
1	254.52 ± 10.15	−20.71 ± 1.65	0.57 ± 0.11
2	201.23 ± 21.89	11.82 ± 0.83	0.28 ± 0.06
3	192.79 ± 17.83	17.03 ± 1.21	0.27 ± 0.04
4	198.77 ± 7.62	18.55 ± 1.37	0.33 ± 0.09

### Endocytic uptake pathway and endosomal escape of the C18-CAMEL/plasmid complexes

**Figure 3.**
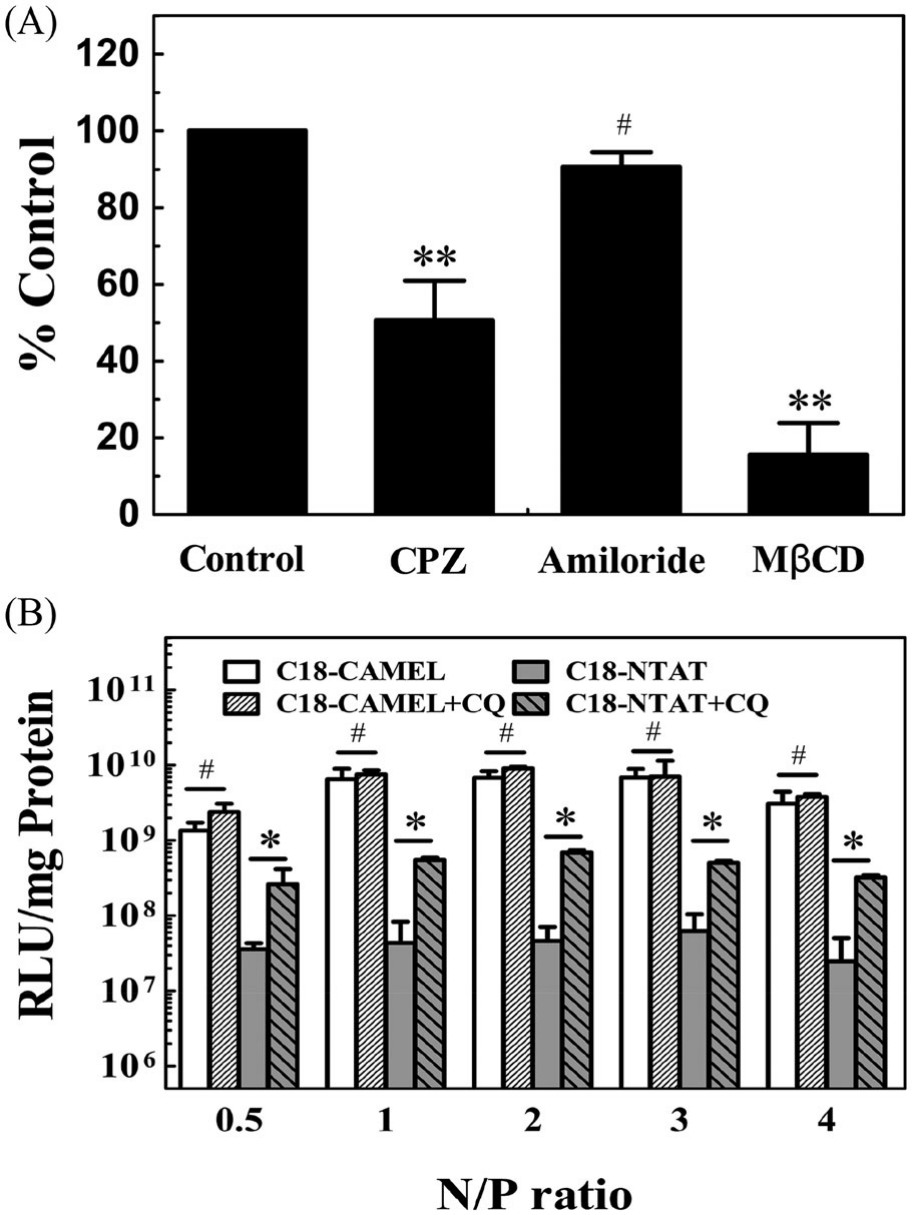
(A) Cellular uptake of C18-CAMEL/Cy5-labeled pGL3 plasmid complexes at an N/P ratio of 2 in the presence of specific endocytosis inhibitors. #*p* > .05 versus control; **p < .01 versus control. (B) Luciferase expression of COS-7 cells treated with C18-CAMEL/pGL3 plasmid complexes in the presence of chloroquine (CQ), C18-NTAT served as the control. #*p* > .05; *p < .05.

Although the cellular uptake mechanism of CPPs remains heavily controversial, there is a general consensus that CPP/nucleic acid complexes enter cells primarily by endocytosis (Margus et al., 2012; Arukuusk et al., 2013; Boisguerin et al., 2015; Lehto et al., 2016). Endocytosis may occur via different types of pathways, such as clathrin-mediated endocytosis, caveolin-mediated endocytosis, and micropinocytosis (Hoyer & Neundorf, 2012; Nakase et al., 2012; Lehto et al., 2016). To further elucidate the endocytic uptake pathways of the C18-CAMEL/pDNA complexes, cells were pretreated with pharmacological endocytosis inhibitors, including chlorpromazine (CPZ) to inhibit clathrin-mediated endocytosis, methyl-β-cyclodextrin (MβCD) to inhibit caveolin-mediated endocytosis, and amiloride to inhibit micropinocytosis (Xiang et al., 2012; Zhang et al., 2013). The results derived from the FACS assay showed that both CPZ and MβCD could reduce the uptake of complexes by 49.4 and 84.5%, respectively (Figure 3(A)). In contrast, amiloride displayed no notable effect on the uptake of complexes. This result demonstrated that the C18-CAMEL/pDNA complexes enter cells by clathrin- and caveolin-mediated endocytosis rather than macropinocytosis. Compared with that of CPZ, the strong inhibitory effect of MβCD indicated that caveolin-mediated endocytosis may be the predominant uptake pathway for the C18-CAMEL/pDNA complexes. Conclusively, our results demonstrated that the C18-CAMEL/pDNA complexes enter cells through endocytosis, and correspondingly, endosomal escape should be considered for efficient transfection.

Efficient endosomal escape is necessary for vector/nucleic acid complexes after internalization through endocytosis. For endosomal escape, attachment of membrane-lytic peptides has proven to be an effective strategy for non-viral vectors (Ferrer-Miralles et al., 2008; Varkouhi et al., 2011; Hou et al., 2015; Komin et al., 2017; Sun et al., 2017). Antimicrobial peptides have been used to enhance the endosomal escape of non-viral gene vectors due to their special membrane-lytic activity (Ferrer-Miralles et al., 2008; Hou et al., 2015). In our previous study, the gene vectors based on the antimicrobial peptide melittin displayed significant endosome-lytic activity (Zhang et al., 2013). Chloroquine (CQ), a well-known endosome-lytic agent, is commonly used to explore whether vector/nucleic acid complexes are trapped in endosomes (Erbacher et al., 1996). In the present study, the coaddition of CQ could only slightly elevate the transfection efficiency of the C18-CAMEL/pDNA complexes at a range of 1- to 2-fold at various N/P ratios (Figure 3(B)). However, the transfection efficiency of C18-NTAT, a control peptide with no membrane-lytic activity (data not shown), substantially increased by 7- to 15-fold at various N/P ratios in the presence of CQ. Taken together, the above results demonstrated that the excellent endosomal escape ability makes C18-CAMEL an ideal gene vector.

### Transfection efficiency of C18-CAMEL in serum and different cancer cell lines

**Figure 4.**
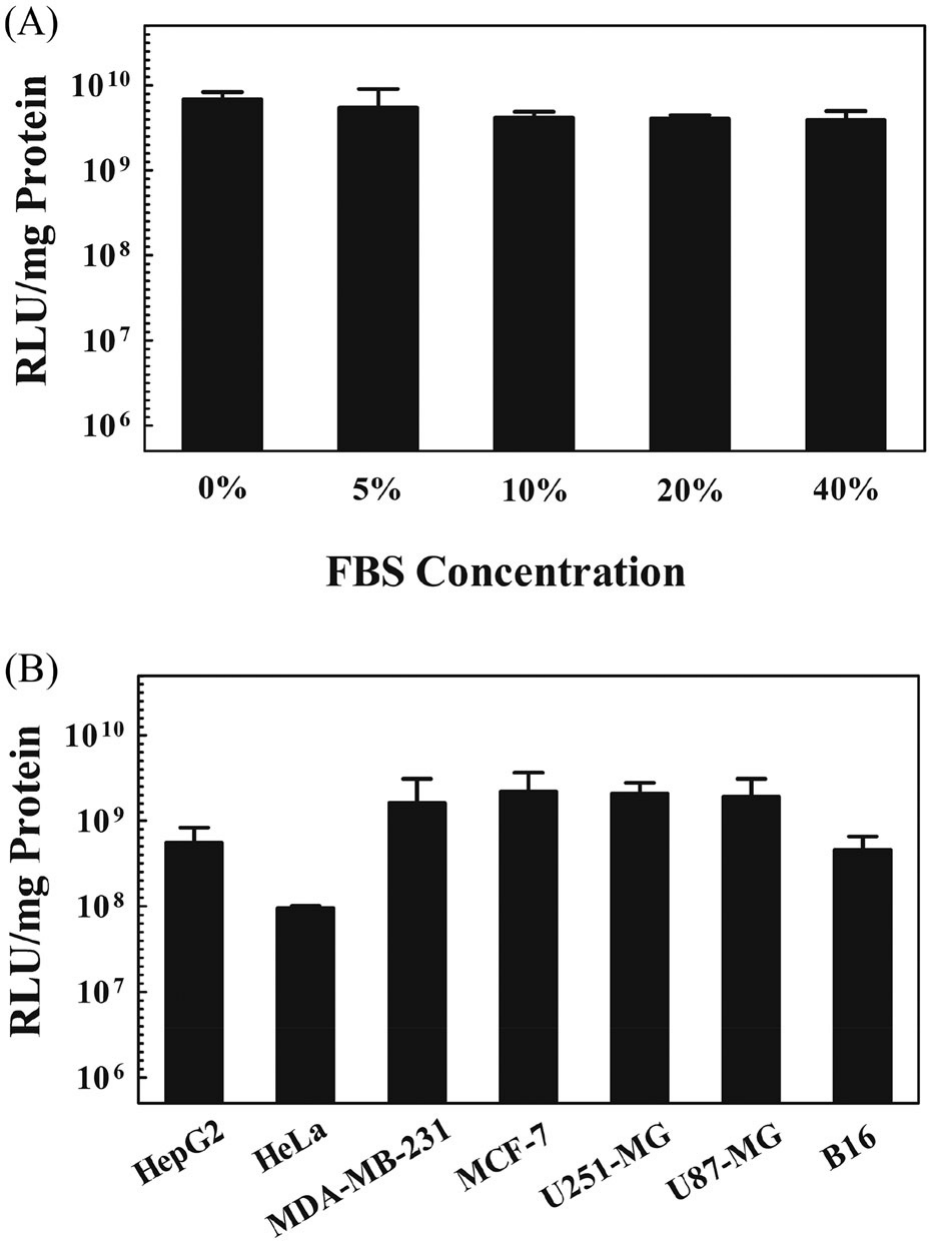
(A) Influence of serum at various concentrations on the luciferase expression of COS-7 cells treated with C18-CAMEL/pGL3 plasmid complexes at an N/P ratio of 2. (B) Luciferase expression of cells treated with C18-CAMEL/pGL3 plasmid complexes at an N/P ratio of 2.

The serum has been described as an important limitation for the transfection efficiency of cationic gene vectors in various ways (Dash et al., 1999; Hoyer & Neundorf, 2012). For example, negatively charged serum proteins can induce aggregation or dissociation of the complexes after nonspecific binding, which substantially hinders cellular uptake and subsequent transfection of complexes (Pack et al., 2005; Hoyer & Neundorf, 2012; Chen J et al., 2019). Therefore, the transfection efficiency of the C18-CAMEL/plasmid complexes at an N/P ratio of 2 in the presence of serum at a range from 5 to 40% was assessed according to a previously reported method (Lehto et al., 2011). As shown in Figure 4(A), the transfection efficiency of C18-CAMEL displayed a slight decrease in the presence of serum. This maybe because C18-CAMEL and pDNA can form stable complexes that are not susceptible to serum (Nguyen et al., 2000; Chen J et al., 2019).

Cell type is considered as an important factor that influences the transfection efficiency of gene delivery vectors. To validate the effectiveness of C18-CAMEL as a non-viral vector for cancer gene therapy, we performed transfection experiments in different cancer cell lines treated with C18-CAMEL/pGL3 plasmid complexes at an N/P ratio of 2. As expected, Figure 4(B) showed that C18-CAMEL exhibited a high transfection efficiency in the tested cancer cell lines, especially breast cancer cell lines (MDA-MB-231 and MCF-7) and glioma cell lines (U87 and U251). Conclusively, C18-CAMEL can transfect several cancer cell lines with satisfactory efficiency.

### Antiproliferative effect of the C18-CAMEL/p53 plasmid complexes

**Figure 5.**
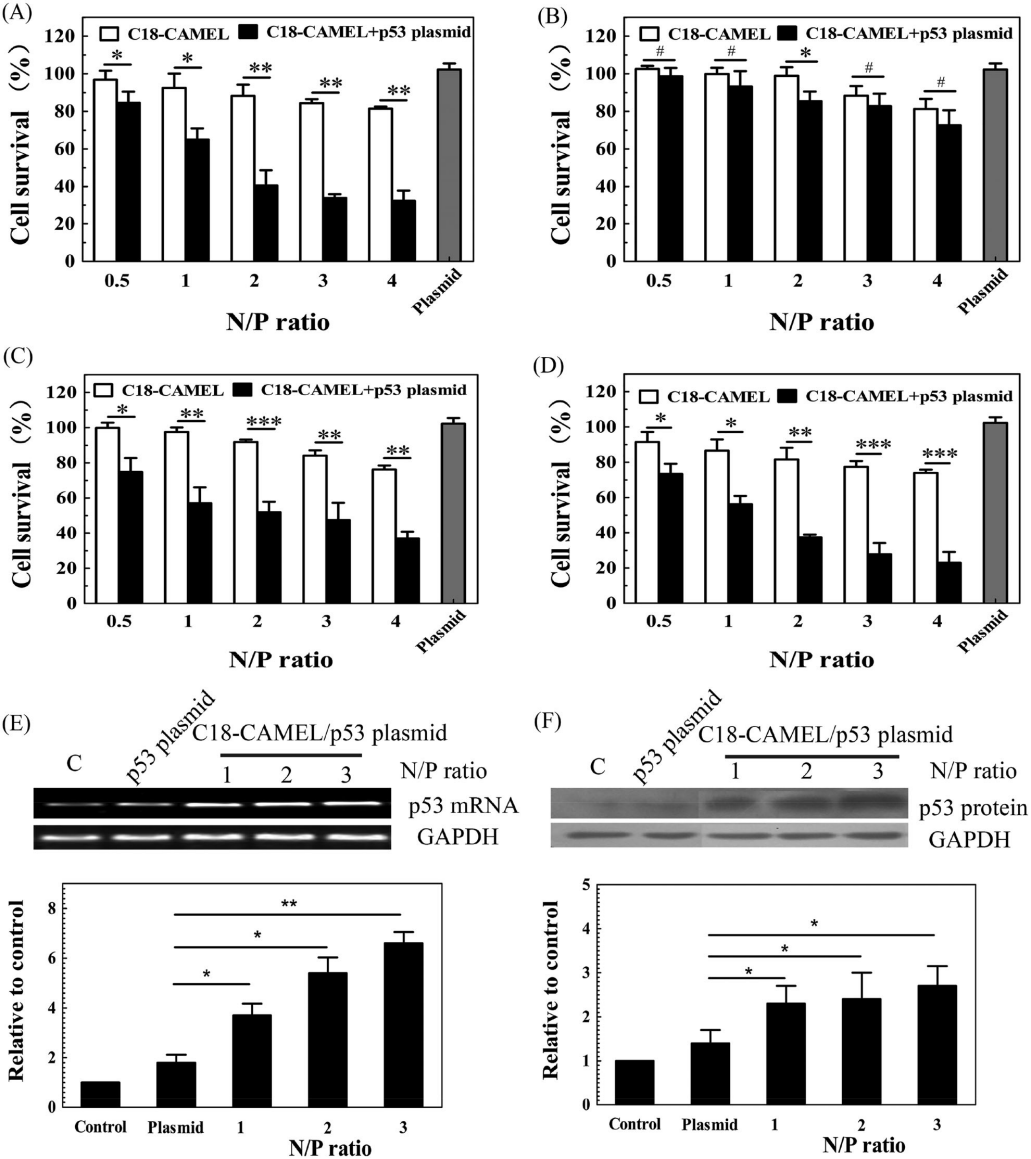
Antiproliferative effects of C18-CAMEL/p53 plasmid complexes against different cell lines. (A) U251 cell line. *p < .05; **p < .01. (B) U87 cell line. ^#^*p* > .05; *p < .05. (C) MDA-MB-231 cell line. *p < .05; **p < .01; ***p < .001. (D) MCF-7 cell line. *p < .05; **p < .01; ***p < .001. (E) p53 mRNA expression of MCF-7 cells treated with C18-CAMEL/p53 plasmid complexes. *p < .05; **p < .01. (F) p53 protein expression of MCF-7 cells treated with C18-CAMEL/p53 plasmid complexes. *p < .05.

The tumor suppressor p53 is an important transcription factor that can trigger cell cycle arrest, cell senescence, and apoptosis. p53 plays a crucial role in preventing tumorigenesis and treating cancer (Cheok et al., 2011; Duffy et al., 2014). However, mutations in the *p*53 gene are one of the most common events and occur in approximately 50% of human cancers (Cheok et al., 2011). In most cancers with no gene mutation, wild-type p53 is always inactivated by several different mechanisms (Wade et al., 2013). Due to the near-universal loss of p53 function in cancers, it can be assumed that restoring functional p53 will suppress tumor growth. Delivery of the wild-type *p*53 gene into cancer cells has proven to be an effective approach for cancer treatment (Cheok et al., 2011; Senzer et al., 2013; Duffy et al., 2014). In order to evaluate the potential of C18-CAMEL in cancer gene therapy, we assessed the anticancer activity of the C18-CAMEL/p53 plasmid complexes against two glioma cell lines (U87 cell line containing the wild-type *p*53 gene; U251 cell line containing a mutant *p*53 gene) and two breast cancer cell lines (MCF-7 cell line containing the wild-type *p*53 gene; MDA-MB-231 cell line containing a mutant *p*53 gene). Our results showed that the C18-CAMEL/p53 plasmid complexes exhibited substantially increased anticancer activity against the mutant p53-expressing U251 cells compared with the cells with the p53 plasmids and C18-CAMEL alone (Figure 5(A)). However, the C18-CAMEL/p53 plasmid complexes displayed slightly increased cytotoxicity against the wild-type p53-expressing U87 cells (Figure 5(B)). This result is in accordance with previous studies showing that mutant p53-expressing glioma cell lines are much more susceptible to p53-based gene treatment than wild-type p53-expressing glioma cell lines (Gomez-Manzano et al., 1996; Shono et al., 2002). In contrast, C18-CAMEL/p53 plasmid complexes displayed high antiproliferative activity against both MDA-MB-231 cells and MCF-7 cells (Figure 5(C,D)). This result demonstrated that the anticancer activity of the C18-CAMEL/p53 plasmid complexes against breast cancer cells does not depend on the p53 status of the cells.

The expression of p53 is a crucial step for p53-based gene therapy. Therefore, to verify whether cell death was attributed to p53 plasmid transfection, we evaluated the p53 mRNA and protein levels in the MCF-7 cells transfected with the C18-CAMEL/p53 plasmid complexes. As shown in Figure 5(E), the p53 mRNA levels of MCF-7 cells transfected with C18-CAMEL/p53 plasmid complexes showed a substantial increase compared with those of the control cells. The MCF-7 cells transfected with naked p53 plasmids did not show obvious changes in the p53 mRNA levels. Subsequently, the results derived from the western blotting assay further confirmed that only the cells transfected with the C18-CAMEL/p53 plasmid complexes displayed a strong increase in the p53 protein expression levels (Figure 5(F)). These results fully confirmed that C18-CAMEL can deliver p53 plasmids into cancer cells, resulting in cell death induced by the expressed p53 protein.

**Figure 6.**
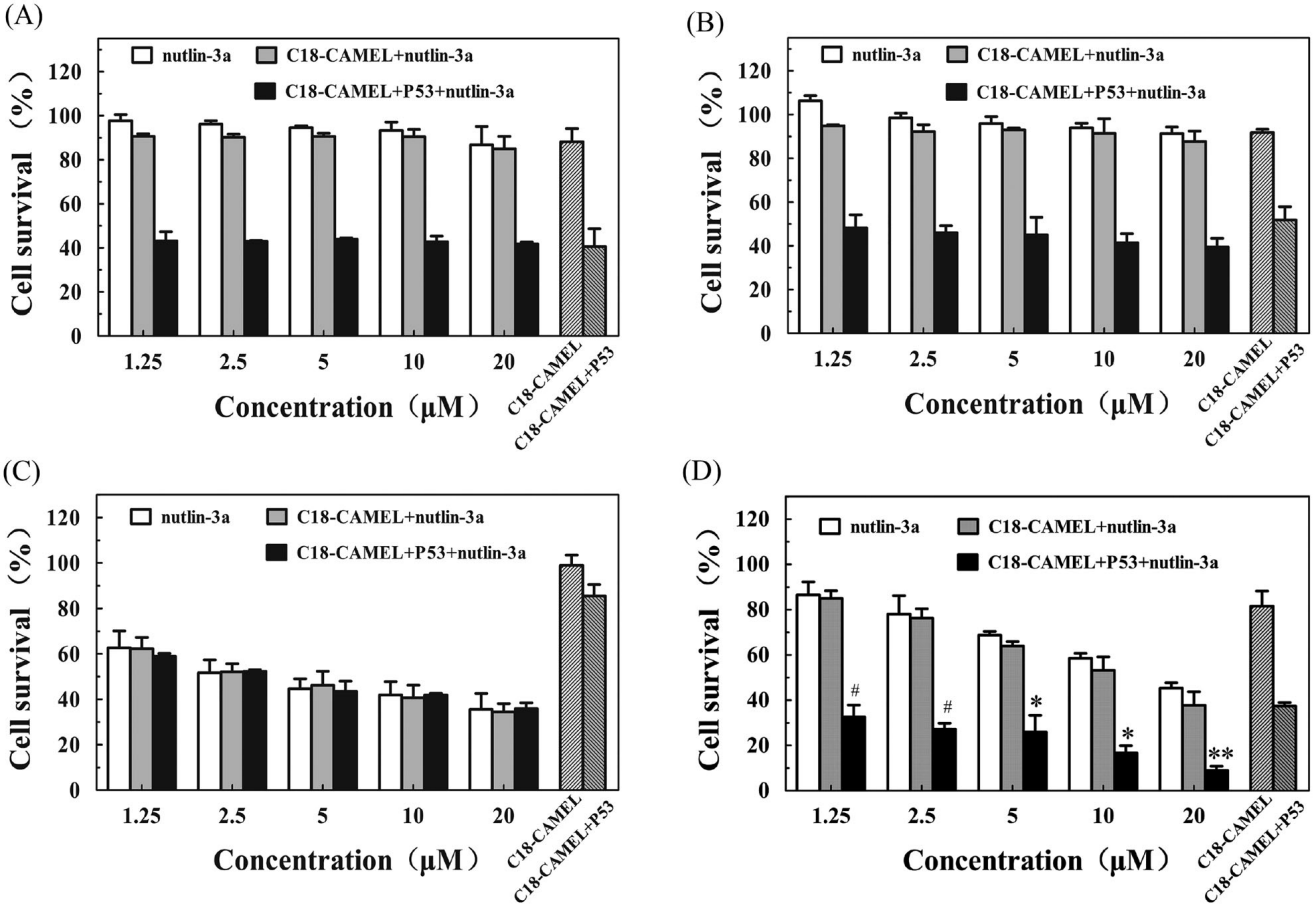
Antiproliferative effects of C18-CAMEL/p53 plasmid complexes at an N/P ratio of 2 in combination with MDM2 inhibitor nutlin-3a against different cell lines. (A) U251 cell line. (B) MDA-MB-231 cell line. (C) U87 cell line. (D) MCF-7 cell line. #*p* > .05 versus C18-CAMEL + P53, *p < .05 versus CAMEL versus C18-CAMEL + P53, **p < 0.01 versus C18-CAMEL + P53.

Because of the pathological complexity of cancer, combined therapy has numerous benefits for cancer therapy. Cancer treatment involving a combination of nucleic acids and drugs has been proven a highly effective strategy (Li et al., 2013; Teo et al., 2016). Murine double minute 2 (MDM2), an E3 ubiquitin ligase that is highly expressed in many cancers, can inactivate wild-type p53 by mediating the nuclear export and degradation of this protein (Wade et al., 2013; Lemos et al., 2016). When the expression levels of p53 increase, the *MDM2* gene will be transcriptionally upregulated accordingly by the negative p53-MDM2 feedback loop, resulting in a decrease in p53 levels (Wade et al., 2013; Lemos et al., 2016). Disruption of the p53-MDM2 feedback loop is a promising approach for restoring p53 activity. Nutlin-3a is a potent and selective MDM2 antagonist that is used for cancer therapy (Lemos et al., 2016). In addition, it was reported that high expression of MDM2 limited the anticancer effect of exogenous p53 (van Beusechem et al., 2005). Therefore, in this study, nutlin-3a was used to treat cancer with the C18-CAMEL/p53 plasmid complexes at an N/P ratio of 2. We assumed that nutlin-3a could protect both endogenous and exogenous p53 from being degraded, resulting in improved therapeutic efficiency. As shown in Figure 6(A,B), nutlin-3a at various concentrations exhibited no obvious anticancer activity against the mutant p53-expressing U251 cells and MDA-MB-231 cells. In addition, nutlin-3a did not significantly increase the anticancer activity of the C18-CAMEL/p53 plasmid complexes against the mutant p53-expressing cells, suggesting that a synergistic antiproliferative effect did not occur. As shown in Figure 6(C), nutlin-3a also did not show synergistic anticancer activity against the wild-type p53-expressing U87 cells with the C18-CAMEL/p53 plasmid complexes despite its high anticancer activity against U87 cells. This result may be related to the low anticancer activity of the C18-CAMEL/p53 plasmid complexes against U87 cells (Figure 5(B)). Gratifyingly, nutlin-3a, and the C18-CAMEL/p53 plasmid complexes displayed significantly synergistic anticancer activity against the wild-type p53-expressing MCF-7 cells (Figure 6(D)). The increased antiproliferative effect of the combined treatment was dependent on the expressed exogenous p53 and MDM2 inactivation by nutlin-3a. Our results confirmed that the combination of an MDM2 inhibitor with p53 gene therapy is an efficient approach for inhibiting the proliferation of cancer cells. However, this combined therapy is not a universal approach for treating all cancer cell lines, and screening cell lines sensitive to this combined therapy is necessary.

## Conclusion

In this study, our results showed that the cellular uptake and transfection efficiency of acyl-CAMEL increased with the increasing chain lengths of conjugated fatty acids. As a hybrid peptide with a short sequence derived from the antimicrobial peptide melittin, C18-CAMEL displayed an enhanced transfection efficiency compared with stearyl-*r*Mel, which was constructed by attaching stearic acid to the retro isomer of melittin and was reported to display high transfection efficiency in our previous study. C18-CAMEL could condense plasmids into stable spherical nanoparticles, which could enter cells by clathrin- and caveolin-mediated endocytosis and escape from endosomes with satisfactory efficiency. More importantly, C18-CAMEL could deliver p53 plasmids into cancer cells and inhibit cell proliferation by expressing p53 protein. In addition, the C18-CAMEL/p53 plasmid complexes and the MDM2 inhibitor nutlin-3a showed synergistic anticancer activity against the wild-type p53-expressing MCF-7 cells, although this combined therapy was not effective in all tested cancer cell lines. Taken together, our study provides an effective strategy for designing efficient and economical non-viral gene vectors based on antimicrobial peptides for cancer gene therapy.

## Supplementary Material

Supplemental Material
