# Supplementary material for: Acylation of the antimicrobial peptide CAMEL for cancer gene therapy
Source: Drug Deliv. 2020 Jul 2;27(1):964–73. doi: 10.1080/10717544.2020.1787556 (PMC8216477; doi:10.1080/10717544.2020.1787556)

Purity analysis of CAMEL, C4-CAMEL, C8-CAMEL, C12-CAMEL, C16-CAMEL, C18-CAMEL, *r*CAMEL, C12-*r*CAMEL, C16-*r*CAMEL and C18-*r*CAMEL was checked by analytical RP-HPLC (Waters), and the peptides were eluted using a liner gradient of 5–95% acetonitrile in 0.1% trifluoroacetic acid at a flow rate of 1mL/min within 45 min on a C18 column.

Solvent:


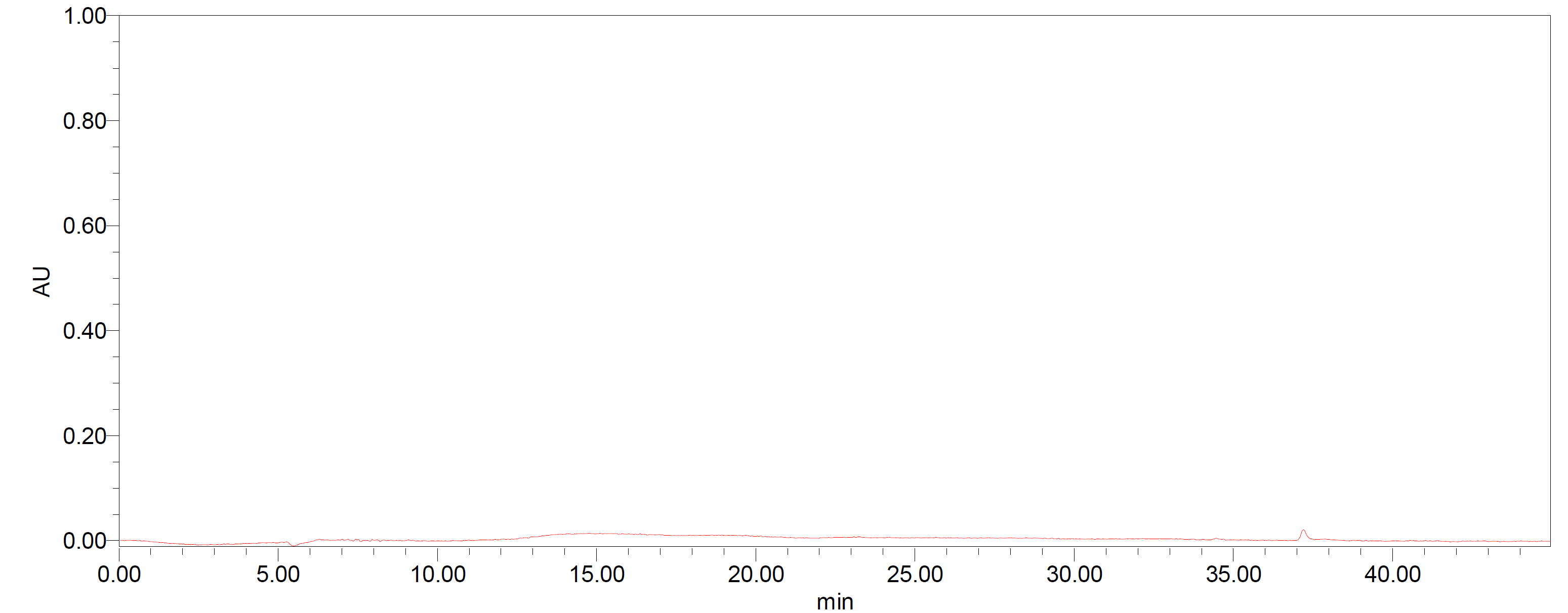


CAMEL:


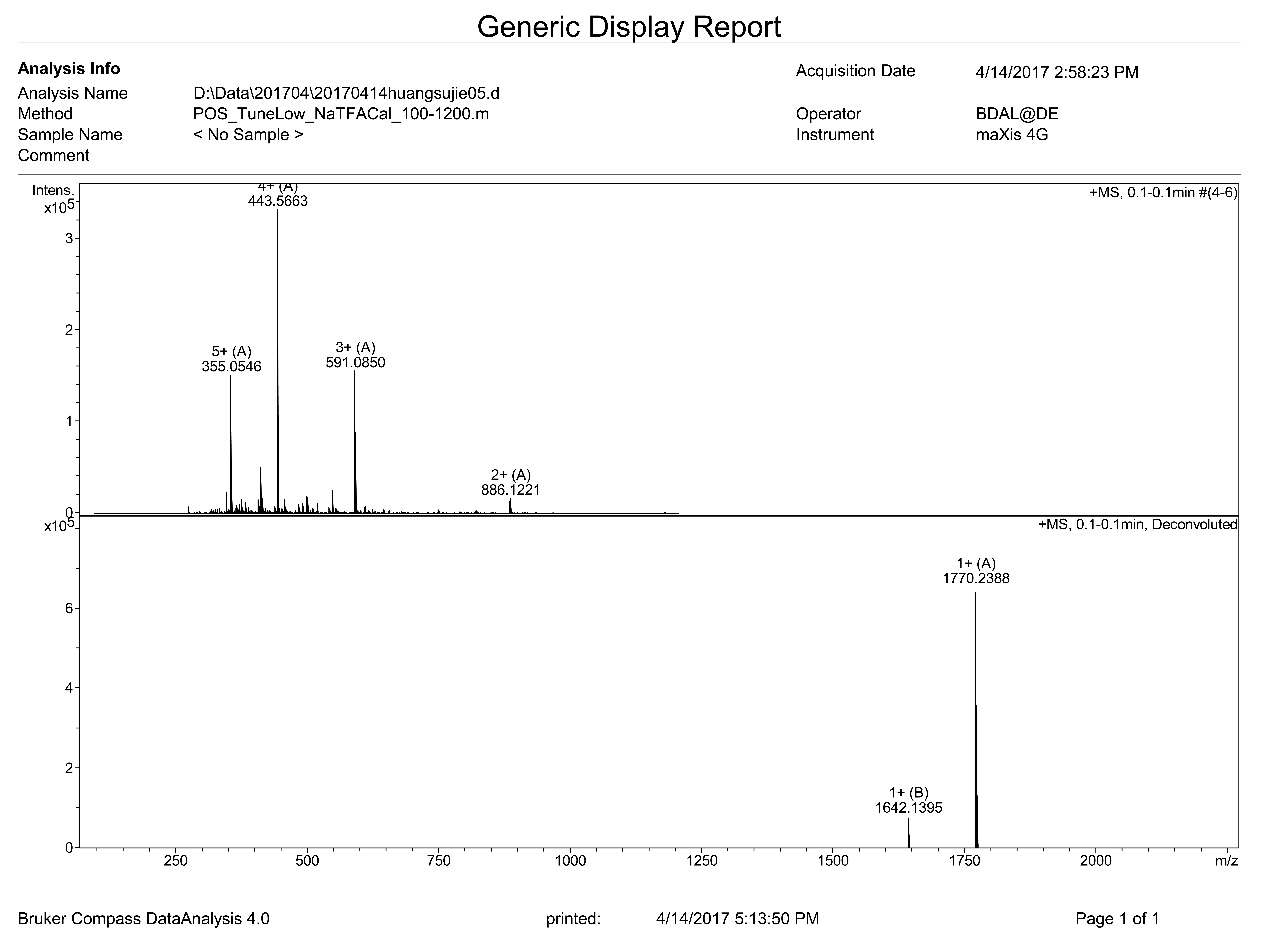


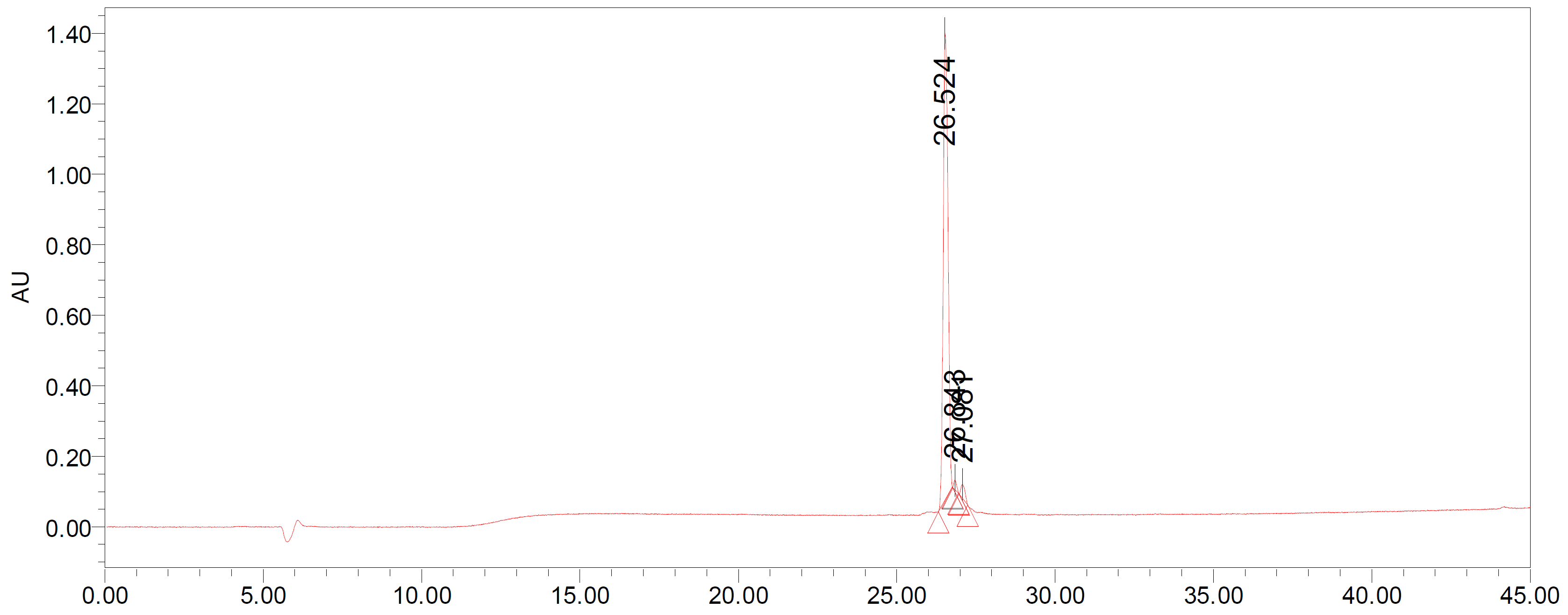


C4-CAMEL


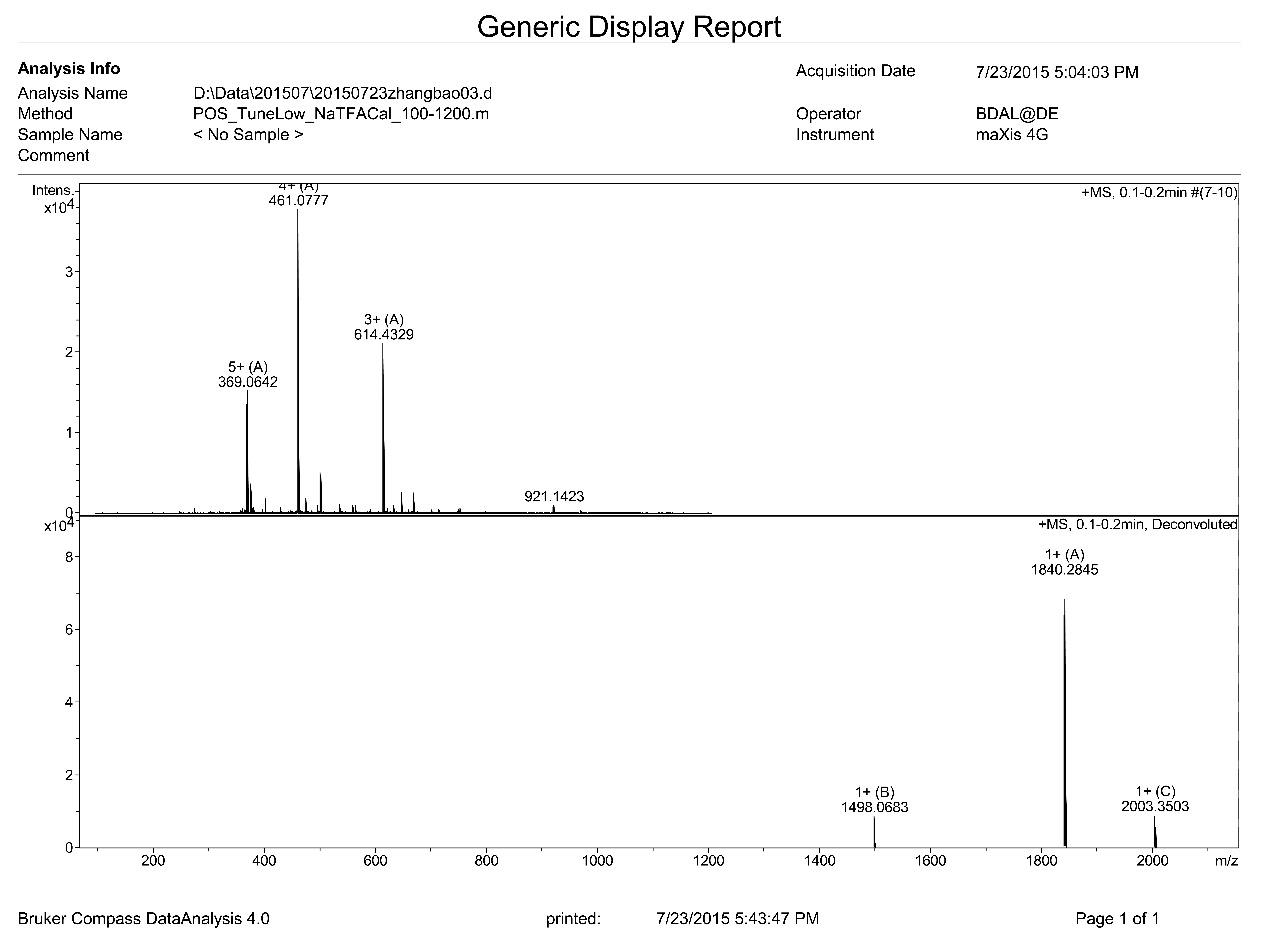


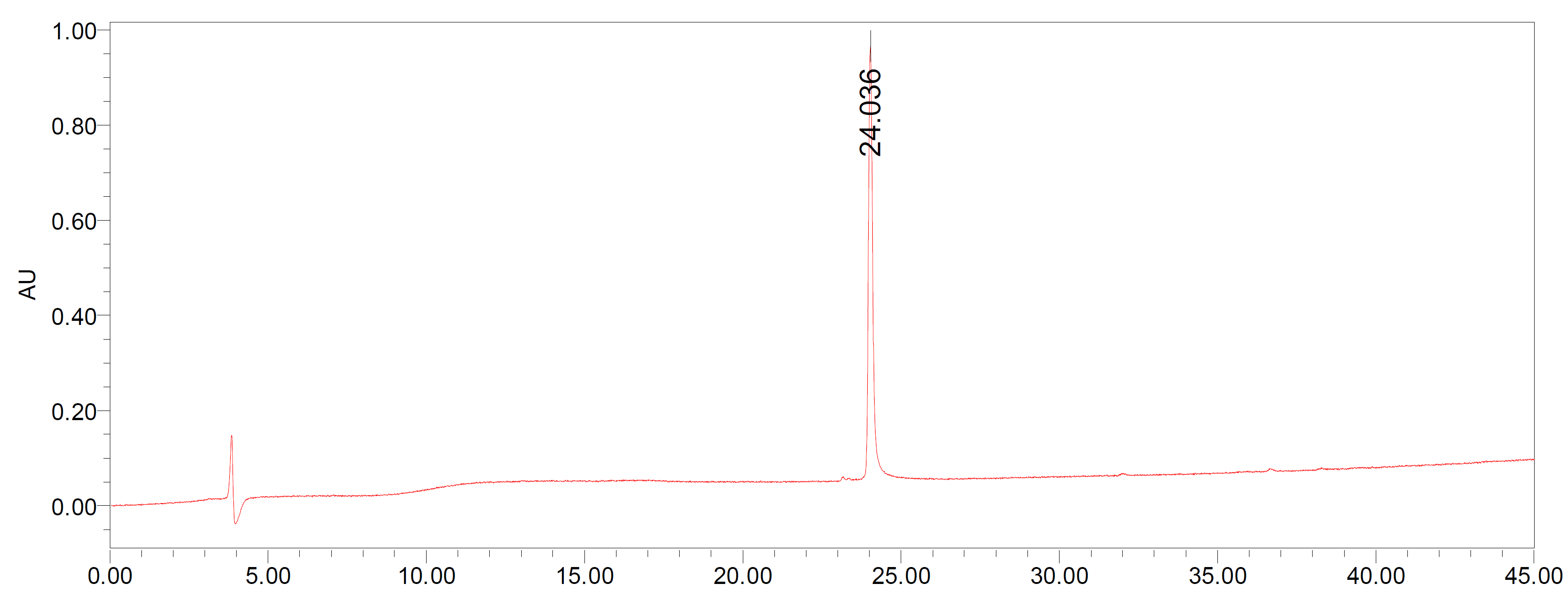


C8-CAMEL


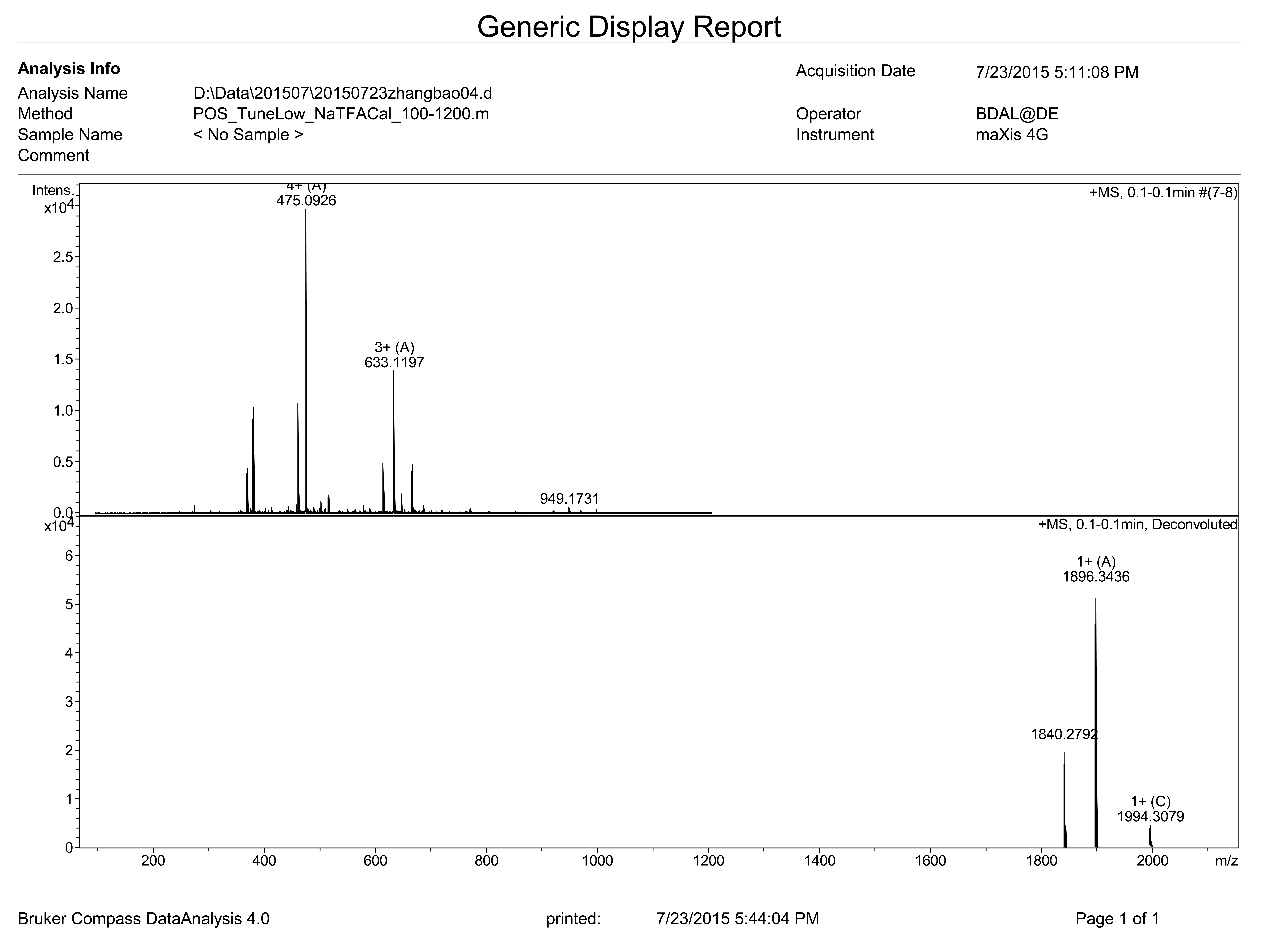


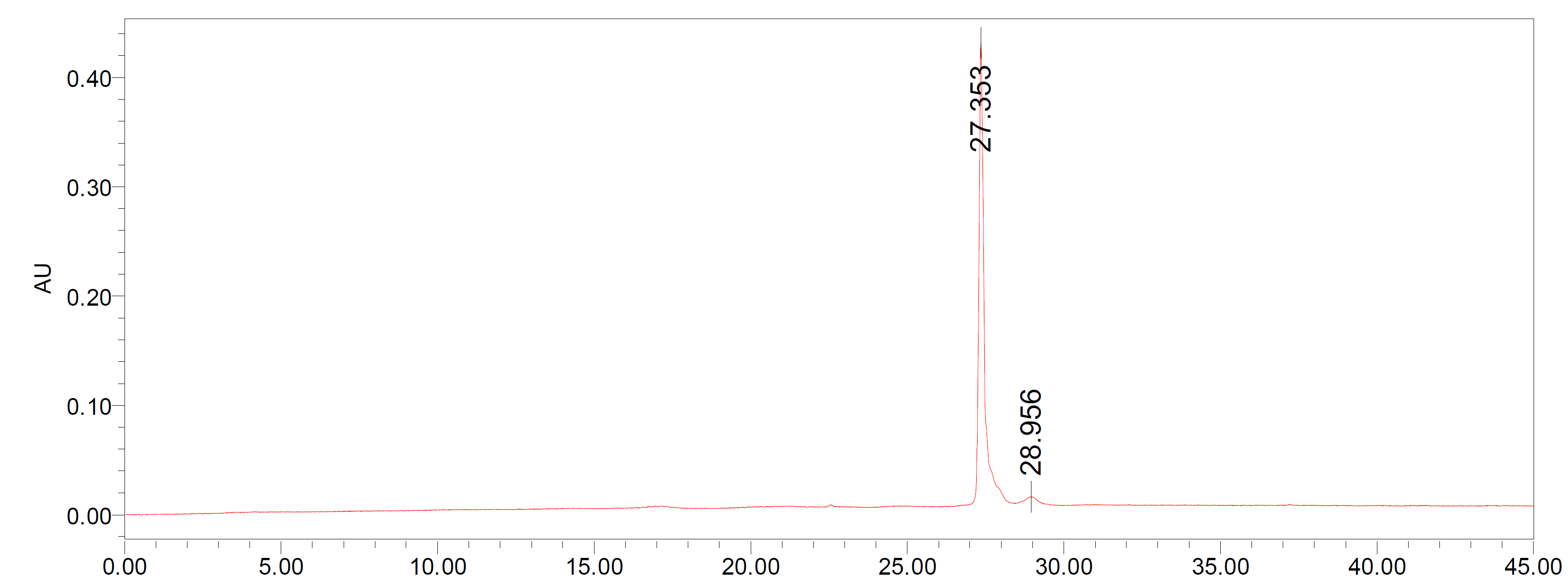


C12-CAMEL


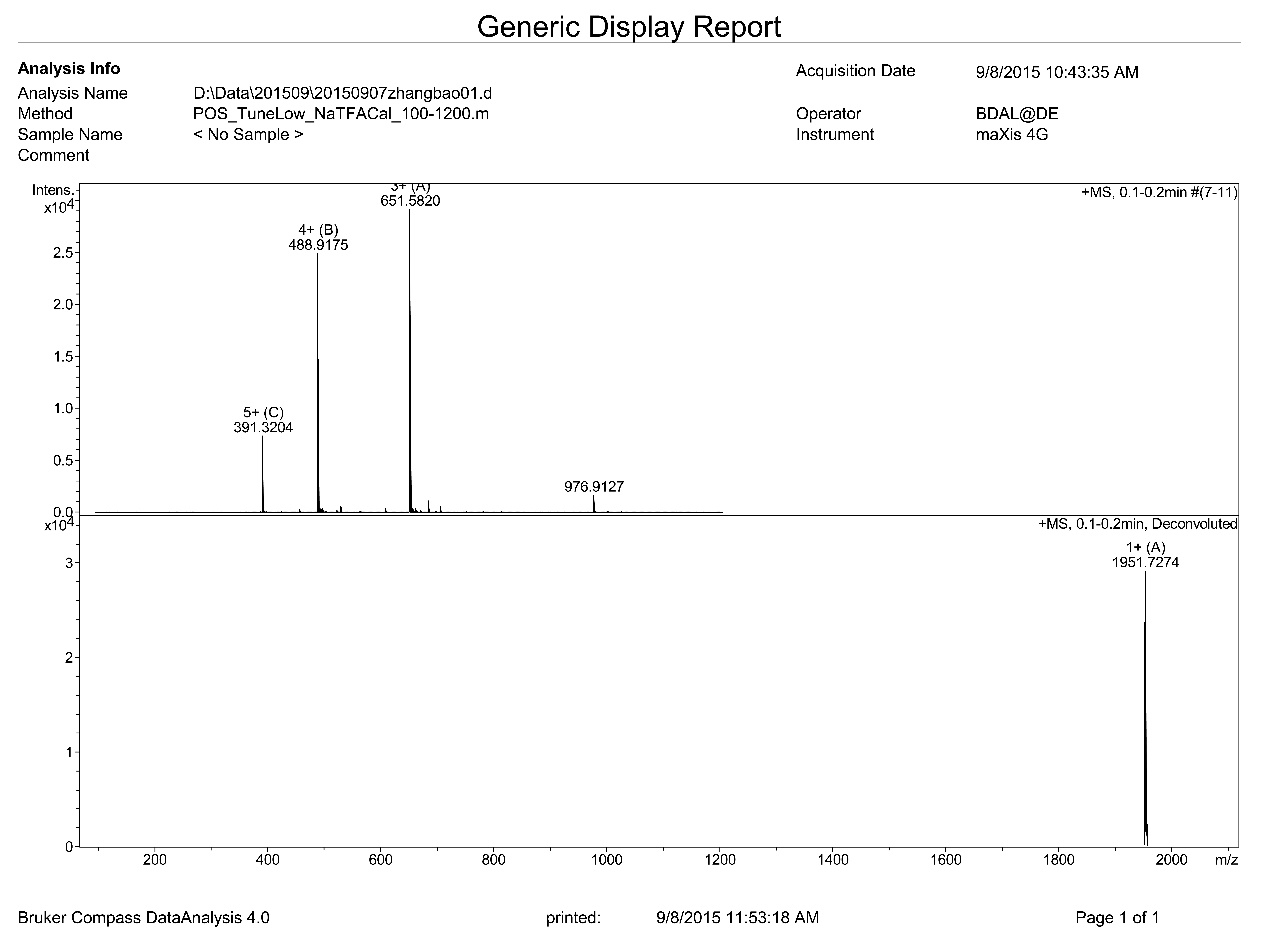


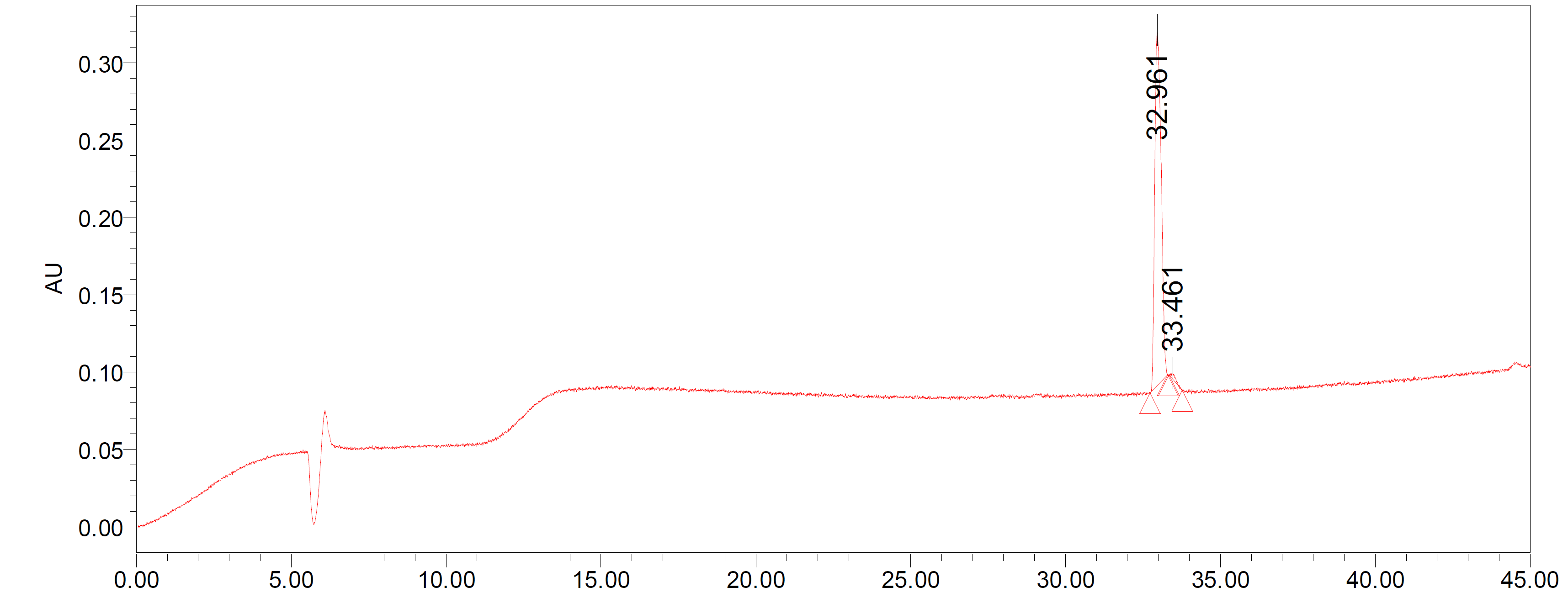


C16-CAMEL


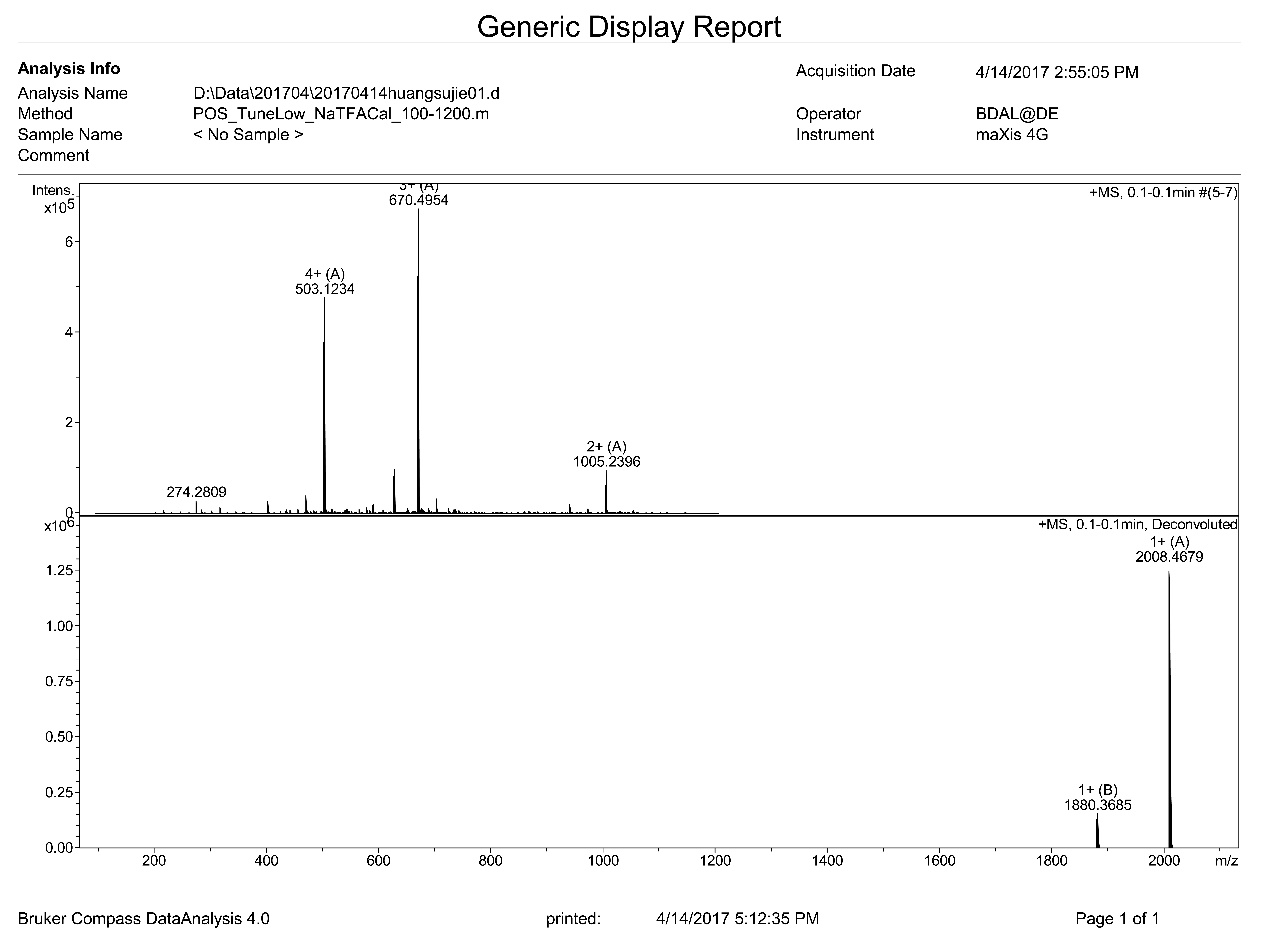


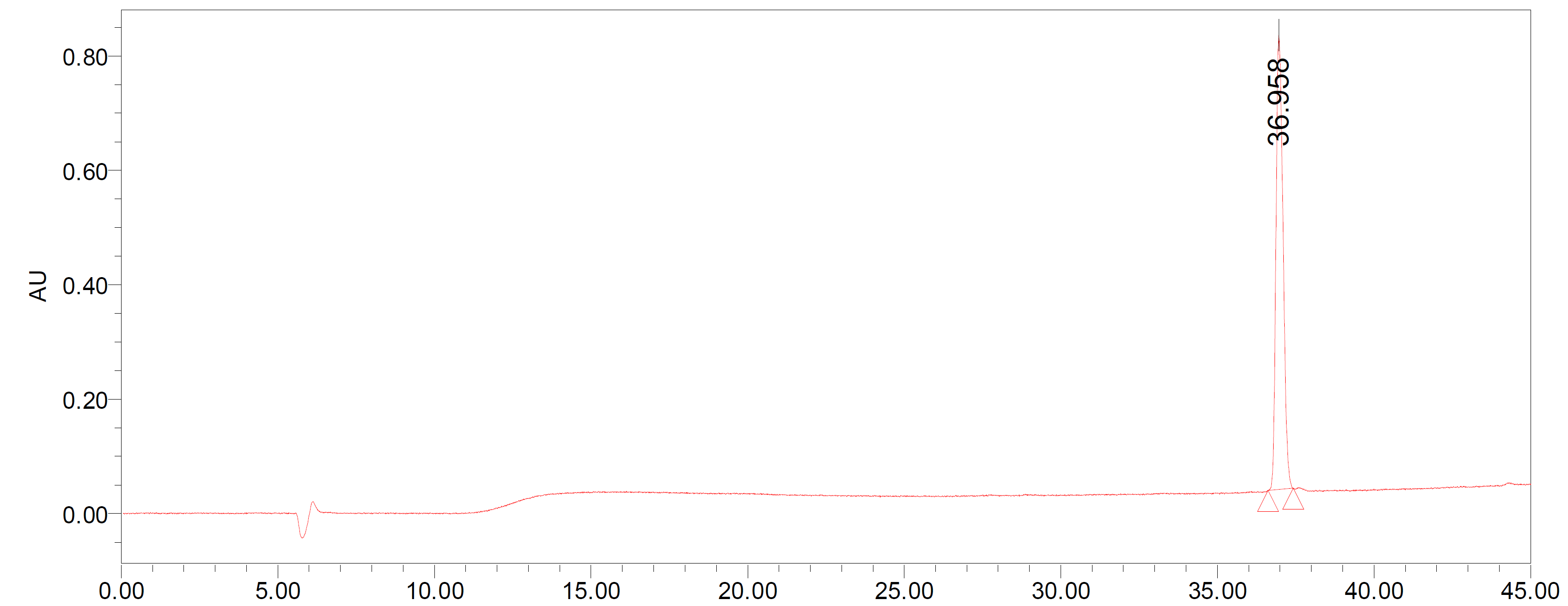


C18-CAMEL


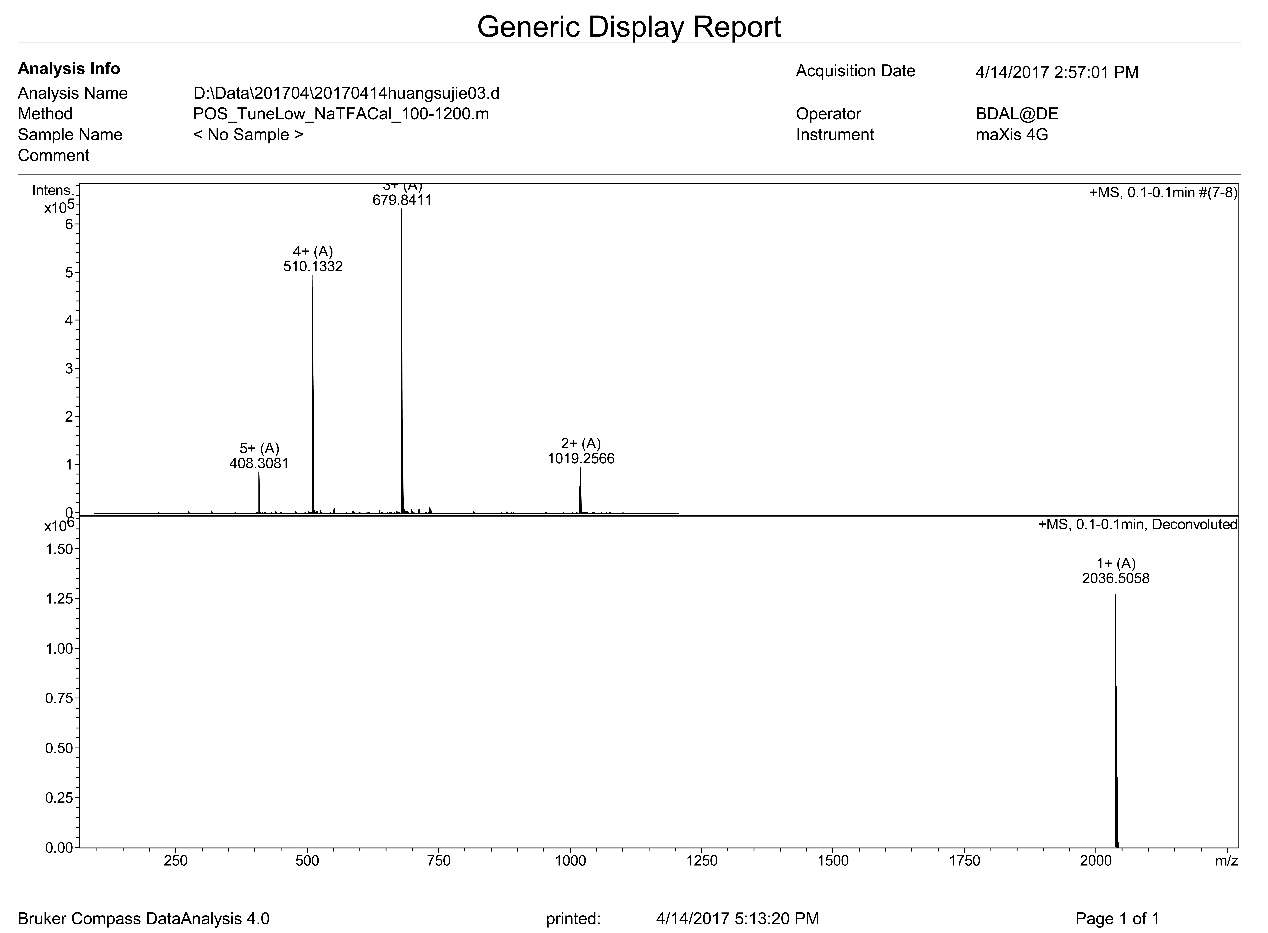


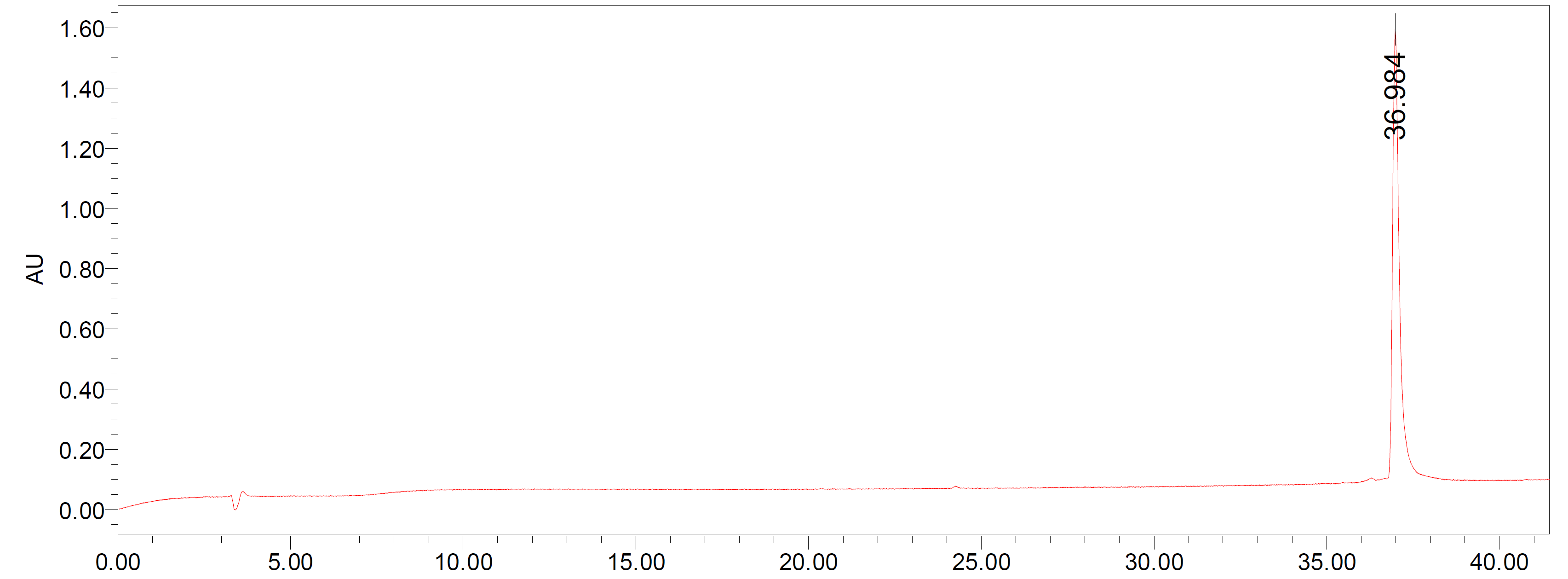


*r*CAMEL


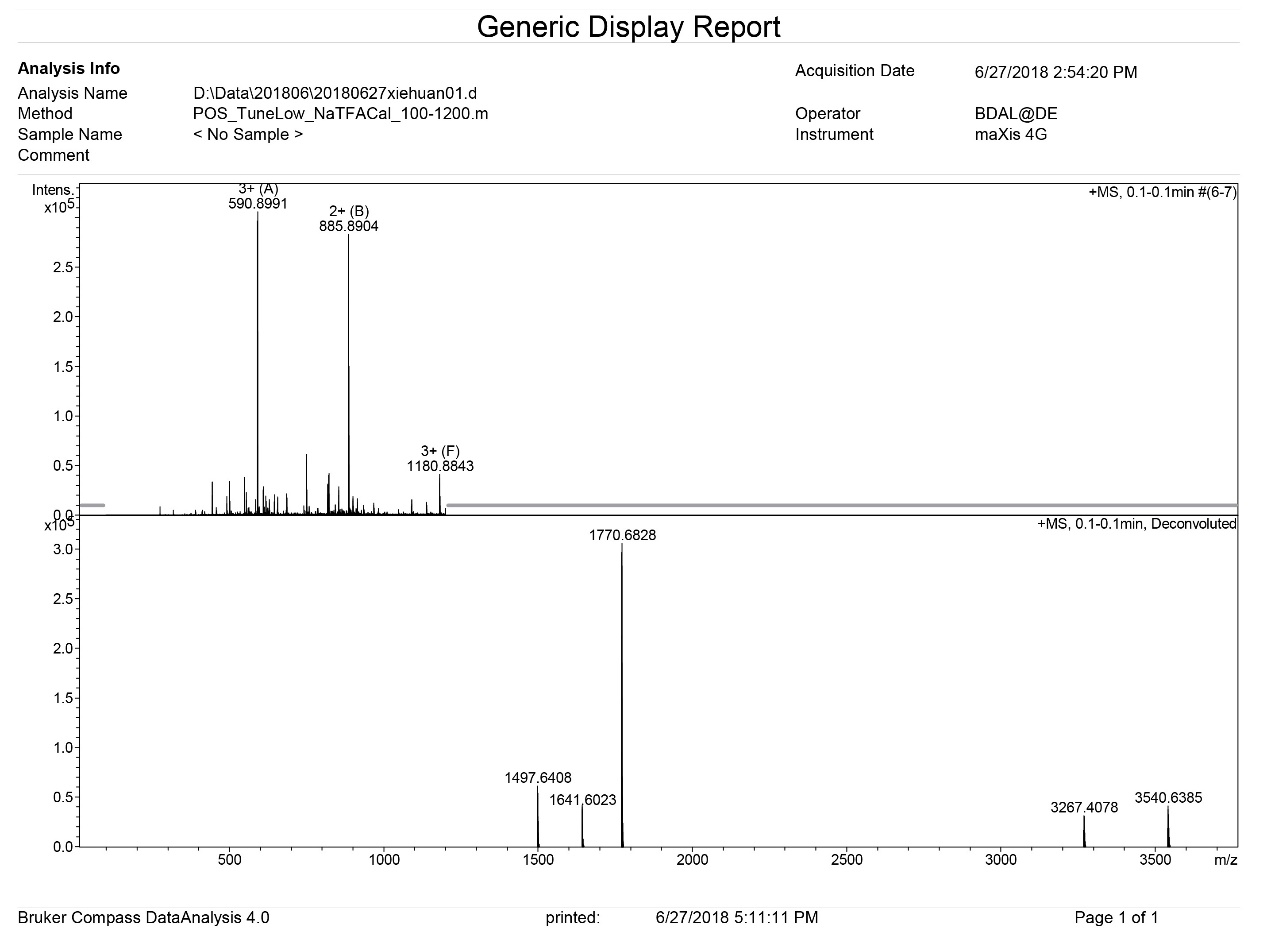

C12-*r*CAMEL




C16-*r*CAMEL




C18-*r*CAMEL




Purity analysis of Stearyl-melittin and C18-NTAT was checked by analytical RP-HPLC (Waters), and the peptides were eluted using a liner gradient of 5–95% acetonitrile in 0.1% trifluoroacetic acid at a flow rate of 1mL/min within 30 min on a C18 column.

Stearyl-melittin





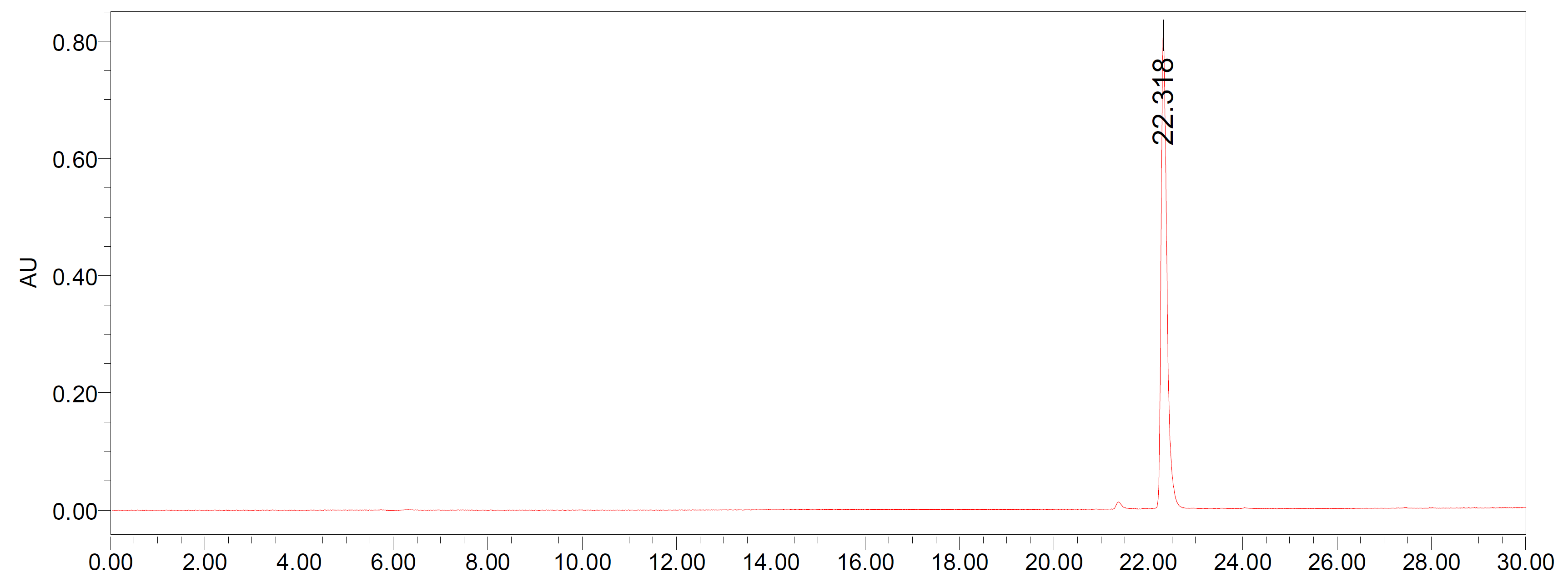


C18-NTAT





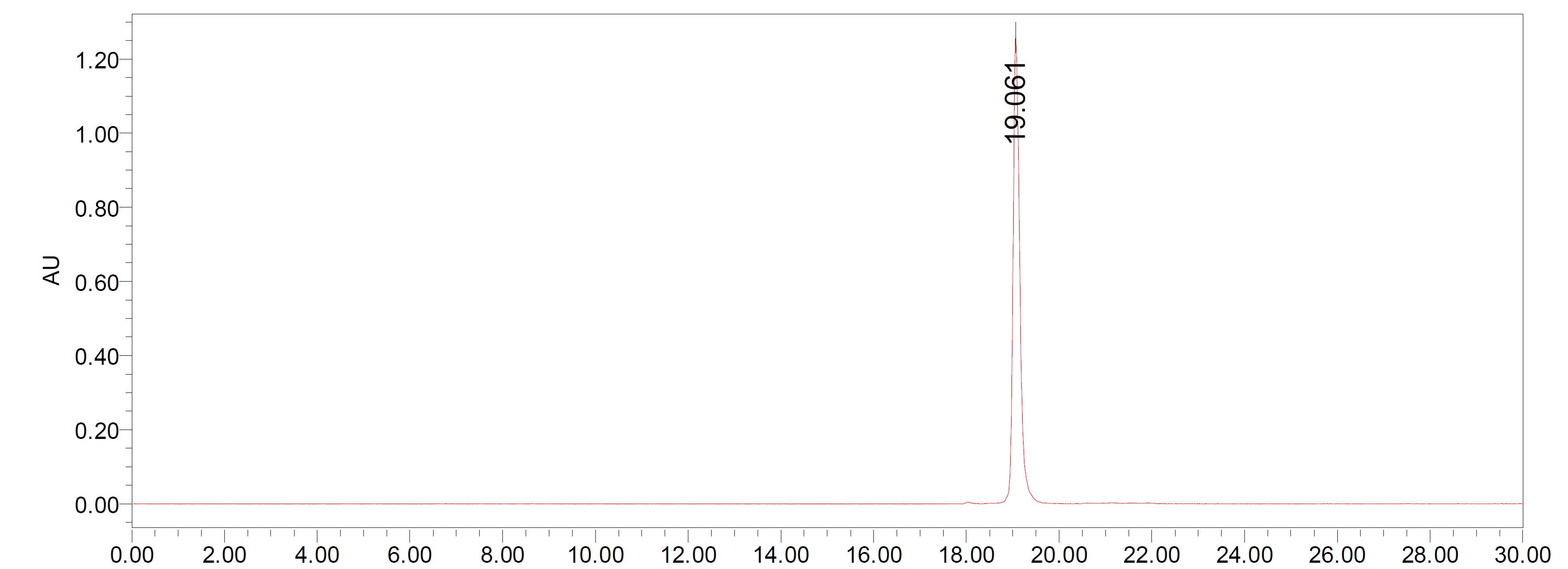

Supplement: Supplemental Material [file IDRD_A_1787556_SM4871.docx]
